# Interpretable machine learning methods for predictions in systems biology from omics data

**DOI:** 10.3389/fmolb.2022.926623

**Published:** 2022-10-17

**Authors:** David Sidak, Jana Schwarzerová, Wolfram Weckwerth, Steffen Waldherr

**Affiliations:** ^1^ Department of Functional and Evolutionary Ecology, Faculty of Life Sciences, Molecular Systems Biology (MOSYS), University of Vienna, Vienna, Austria; ^2^ Department of Biomedical Engineering, Faculty of Electrical Engineering and Communication, Brno University of Technology, Brno, Czech Republic; ^3^ Vienna Metabolomics Center (VIME), Faculty of Life Sciences, University of Vienna, Vienna, Austria

**Keywords:** multi-omics, interpretable machine learning, deep learning, explainable artificial intelligence, metabolomics, proteomics, transcriptomics

## Abstract

Machine learning has become a powerful tool for systems biologists, from diagnosing cancer to optimizing kinetic models and predicting the state, growth dynamics, or type of a cell. Potential predictions from complex biological data sets obtained by “omics” experiments seem endless, but are often not the main objective of biological research. Often we want to understand the molecular mechanisms of a disease to develop new therapies, or we need to justify a crucial decision that is derived from a prediction. In order to gain such knowledge from data, machine learning models need to be extended. A recent trend to achieve this is to design “interpretable” models. However, the notions around interpretability are sometimes ambiguous, and a universal recipe for building well-interpretable models is missing. With this work, we want to familiarize systems biologists with the concept of model interpretability in machine learning. We consider data sets, data preparation, machine learning methods, and software tools relevant to omics research in systems biology. Finally, we try to answer the question: “What is interpretability?” We introduce views from the interpretable machine learning community and propose a scheme for categorizing studies on omics data. We then apply these tools to review and categorize recent studies where predictive machine learning models have been constructed from non-sequential omics data.

## 1 Introduction

Machine learning (ML) is advancing rapidly, with new methods introduced almost daily. As the field progresses, also its methods become better accessible to researchers from other disciplines due to the development and release of new software tools. Many fundamental ML methods can be applied to almost any data set. Nonetheless, the real-world goals of researchers that apply these methods to their own data sets may diverge from the objectives of the ML model itself (Lipton, 2016). While a researcher may want to understand the molecular mechanisms of a disease or may want to know why a ML model classifies a patient as having a disease, the ML model may aim to minimize the number of wrong predictions. Understanding predictions is especially important in a clinical context, where medical professionals need to justify healthcare decisions (Barredo Arrieta et al., 2020). Bringing real-world and ML objectives into harmony asks for methods that make ML models more interpretable (Lipton, 2016). The research field behind this goal is *interpretable machine learning* (Murdoch et al., 2019), which falls under the umbrella of *explainable artificial intelligence (XAI)* (Barredo Arrieta et al., 2020). Advances in this domain are becoming even more important as ML models are increasing in complexity. Further, using data-driven approaches like machine learning to not just predict from data but also to learn about the biological mechanisms that generate the data in the first place is an attractive concept. Mechanistic approaches like kinetic models take long to develop and require a detailed prior understanding of a system, while machine learning models can make better predictions and sometimes answer the same biological questions with less effort (Costello and Martin, 2018).

Consequently, interpretable ML has received more and more attention in biology in recent years. Various studies that apply machine learning to biological data sets have been published, many claiming to implement “interpretable” (Wang et al., 2020; Oh et al., 2021; Sha et al., 2021), “explainable” (Manica et al., 2019), “gray-box” (Nguyen et al., 2021), “white-box” (Yang et al., 2019) or “visible” (Ma et al., 2018) machine learning frameworks. All these terms refer to the urge to gain valuable biological knowledge from data with the help of machine learning, which falls under the keyword “interpretability” (Lipton, 2016; Murdoch et al., 2019). Now, the question arises, what is interpretability?, or, more specifically, what makes a machine learning model interpretable? The answer to this fundamental question is under debate in the machine learning community for some time now. Many answers have been proposed (Lipton, 2016; Murdoch et al., 2019; Barredo Arrieta et al., 2020), but a clear consensus is still missing. Generally, “interpretability [itself] is a broad, poorly defined concept (Murdoch et al., 2019),” which is probably the main reason why definitions in a machine learning context are complicated to fix. Clearly, there are different perspectives to view interpretability in machine learning: e.g., it can mean how much we can learn from data by using a ML model (Murdoch et al., 2019), how well we understand the ML model itself (i.e., comprehend how it makes a prediction), or how much extra information the model can provide that supports predictions (Lipton, 2016). *Interpretation methods*, the techniques by which we gain biological insight from data with machine learning besides predictions, may divide into “model-based” and “post hoc” methods (Murdoch et al., 2019). While model-based methods rely on adapting the model before training it, post-hoc methods operate on already trained models (Murdoch et al., 2019).

In machine learning, there are three main ways to train models, namely reinforcement learning, unsupervised learning, and supervised learning. Throughout this review, we want to focus on *supervised learning* because of its prevalence in general (LeCun et al., 2015) and in the context of predictive systems biology. Supervised learning presents models with a set of training *samples* (e.g., omics profiles from multiple patients) for which the outcome of a prediction (e.g., health conditions) is already known (Presnell and Alper, 2019). Based on this training data set, supervised learning tries to produce a model that accurately predicts the target for samples without a known solution (Angermueller et al., 2016). Supervised machine learning techniques have been applied to high-throughput omics data to predict a broad range of clinical, phenotypical, and physiological observations.

While diagnosing various diseases (Leitner et al., 2017; Trainor et al., 2017; Hu et al., 2018; Pai et al., 2019; Stamate et al., 2019; Nguyen et al., 2021; Sha et al., 2021; van Dooijeweert et al., 2021) or predicting clinical outcomes (Bahado-Singh et al., 2019; Pai et al., 2019; Zhang et al., 2021) seem common, possible applications reach up to inference of the fluxome (Alghamdi et al., 2021) or growth rate (Culley et al., 2020) of a cell from transcript levels. Besides using machine learning for predictions, many studies attempt to gain additional biological knowledge by implementing post-hoc or model-based interpretation methods (Alakwaa et al., 2018; Date and Kikuchi, 2018; Hu et al., 2018; Bahado-Singh et al., 2019; Wang et al., 2020; Nguyen et al., 2021; Wang et al., 2021). Further, interpretability can improve by incorporating prior biological knowledge into a research project (Nguyen et al., 2021; Wang et al., 2021).

This review was written from an interdisciplinary perspective and is intended for an audience with systems biological background but not necessarily experience in machine learning, who are interested in machine learning approaches for generating biological insight. We aim to familiarize readers with the term interpretability and equip them with a fundamental machine learning background necessary for understanding the concept. To achieve this, we take an example-based approach by highlighting studies that successfully extract biological insight from non-sequential omics data sets with the help of interpretation methods.

Furthermore, we present a scheme for categorizing research papers based on two criteria, 1) the use of interpretation methods and 2) at which point prior knowledge enters a research project. With this categorization system, we hope to contribute to the establishment of terms associated with interpretability and allow ML projects to be compared in their interpretability. In this work, we have assigned a total of 26 publications to 9 categories that our scheme outlines.

We start with a characterization of the utilized data sets, what studies predict from them, and how to prepare them for machine learning. Then we present supervised learning methods that systems biologists applied to omics data and showcase available software tools for data manipulation, visualization, and up to fully automatic ML solutions for omics data analysis. We try to answer the question: “What is interpretability?” by introducing fundamental concepts, describing our categorization scheme, and highlighting exemplary works in systems biology. With this work, we want to raise awareness for interpretable machine learning and its potential for gaining insight from omics data.

## 2 Data sets

Due to the *data-driven* nature of machine learning, data is essential for a successful ML project (Mendez et al., 2019). Ultimately, any machine learning model tries to learn discriminative features, relationships, patterns, or structures found within a data set. In a data set for supervised learning, a sample consists of variables that describe its properties (the *features*, e.g., molecule abundances) and has one or more outcome variables associated with it that provide the corresponding prediction target (the *labels*) (Shalev-Shwartz and Ben-David, 2013; Angermueller et al., 2016; Deisenroth et al., 2020). Labels can be any variables we wish to predict, ranging from categorical variables describing cancer (sub)types (Alakwaa et al., 2018; Sharma et al., 2019; Zhang et al., 2021) to continuous specifications of cell growth (Kim et al., 2016; Culley et al., 2020). Based on whether labels are categorical or quantitative variables, one differentiates between the two supervised prediction tasks, *classification* or *regression* (Bishop, 2006, p. 3). A feature can be any variable we expect to be predictive of a target variable, such as metabolite abundances (Trainor et al., 2017; Stamate et al., 2019; Sha et al., 2021), “traditional risk factors” (Liu et al., 2017), metabolic fluxes (Culley et al., 2020), and even kinetic parameters when the goal is to predict the feasibility of kinetic models (Andreozzi et al., 2016). Samples with known labels provide the “ground truth” enabling the ML model to learn how predictions for unlabeled samples should optimally look like (Martorell-Marugán et al., 2019).

Usually, the data set that holds all collected and labeled samples is divided into at least a *training set* and an independent *test set* (Trainor et al., 2017; Alakwaa et al., 2018; Sharma et al., 2019; Culley et al., 2020; van Dooijeweert et al., 2021). A *learning algorithm* uses the training set to improve/construct a ML model (Bousquet and Elisseeff, 2002), e.g., by estimating parameters or functional forms. Since the model is fit to the training data, the model’s error on this data can be drastically smaller on unseen data like the test set (Maceachern and Forkert, 2021), which means that the model struggles on new samples drawn from the same underlying distribution, i.e., the model has a poor “generalization” ability (Shalev-Shwartz and Ben-David, 2013, sect. 1.1). This phenomenon is known as *overfitting*. Guiding high-level modeling decisions (i.e., *hyperparameters* like the number of layers in a neural network) with the test set can similarly overfit the model to this data (Bishop, 2006, p. 32). It is, therefore, required to use a third separate *validation set* (Angermueller et al., 2016) or, if samples are rare, use other techniques like cross-validation that avoid using the test set for such optimization purposes (Bishop, 2006, p. 32f). After tuning the design and training, a model’s realistic *performance*, i.e., “predictive accuracy” (Murdoch et al., 2019) is measured on the out-of-sample test set (Angermueller et al., 2016).

With omics data sets becoming more readily available, they are also more frequently exposed to machine learning algorithms. Alone in this review, the categorized studies covered eight distinct data types characterizing a biological system—not counting network-type data. Omics data sets lend themselves to interpretable machine learning solutions because of their sheer complexity, making them hard to interpret by visual inspection or simple statistical methods. Table 1 provides an overview of the reviewed studies that demonstrates a wide diversity of prediction targets. We compile some of the targets into the categories “Diagnosis,” “Clinical Outcome,” and “Physiology.” Physiology includes phenotypic predictions, genetic properties, cellular state and dynamics, etc. Predictions that did not fit any of these categories were regional origin of an organism (Date and Kikuchi, 2018), type of a cell (Wang et al., 2020; Wang et al., 2021), “feasibility” of kinetic models (Andreozzi et al., 2016), and body region where a tumor emerged (Zhang et al., 2021). The most common category was Diagnosis with 16 examples. Among the diagnosed diseases, cancer is most prevalent. One reason is the commendable availability of large omics data sets enabled by The Cancer Genome Atlas (TCGA) program. Unarguably, precision medicine, especially cancer research and diagnostics has benefited a lot from machine learning in recent years (Grapov et al., 2018; Chiu et al., 2020). Another trend that seems to arise is the application of machine learning to problems that have been traditionally solved with mechanistic models, like the estimation of metabolic fluxes (Alghamdi et al., 2021) and metabolite changes over time (Costello and Martin, 2018). Phenotypic discrimination is also very apparent. This includes predicting cell growth (Kim et al., 2016; Culley et al., 2020), patient biological sex (Zhang et al., 2021), and organism body size (Asakura et al., 2018). Zhang et al. (2021) demonstrated that even multiple predictions, ranging from cancer type classification and stratification over patient age and sex to patient survival, are possible from the same integrated data source. Building large “multi-task” (Zhang et al., 2021) machine learning frameworks that can predict multiple biological system properties for one sample seem promising as data collections grow and become more well-curated, as exemplified by Kim et al. (2016).

**TABLE 1:** Overview of the categorized studies.

Omics data type	Prediction Method(s)	Effective raw features	Effective raw samples	Prediction type	Ref
Metabolomics	**Ensemble DNN,** DNN, RF, SVM	106 NMR peaks	502 profiles	Regression (Physiology; fish body size)	Asakura et al. (2018)
Metabolomics	LogReg	24 metabolites	1571 profiles	Binary Classification (Clinical Outcome; prospective type 2 diabetes)	Liu et al. (2017)
Metabolomics	SVM	1737 metabolites	58 profiles	Binary Classification (Diagnosis; Diamond Blackfan Anaemia)	van Dooijeweert et al. (2021)
Metabolomics	**RF**, AdaBoost, SVM, NBC	109 metabolites	12–18^a^ profiles	Binary Classification (Physiology; pathway presence in tomato pericarp)	Toubiana et al. (2019)
Metabolomics	DNN, XGBoost (DT), RF	347 metabolites	357 profiles	Binary Classification (Diagnosis; alzheimer-type dementia)	Stamate et al. (2019)
Metabolomics	**LGP**, LogReg	70 metabolites	389 profiles	Binary Classification (Diagnosis; knee osteoarthritis)	Hu et al. (2018)
Metabolomics	**LGP**, SVM, RF	242 metabolites	114–115 profiles	Binary Classification (Diagnosis; alzheimer’s disease, amnestic mild cognitive impairment)	Sha et al. (2021)
Metabolomics	**DNN**, PLS-DA, RF, SVM	≤106 NMR peaks	1022 profiles	Binary Classification (Other; regional origin of fish)	Date and Kikuchi (2018)
Metabolomics	PLS-DA, Sparse PLS-DA, RF, SVM, kNN, NBC, ANN	≤1032^b^ metabolites	38 profiles	Multi-class Classification (Diagnosis; cardio vascular disease)	Trainor et al. (2017)
Metabolomics	PLS-DA, Sparse PLS-DA, RF, SVM, kNN, NBC, ANN	≤431^b^ metabolites	not assigned^a^	Binary Classification (Diagnosis; adenocarcinoma lung cancer)	Trainor et al. (2017)
Metabolomics	PLS-DA, Sparse PLS-DA, RF, SVM, kNN, NBC, ANN	not assigned^a^	not assigned^a^	Multi-class Classification (Physiology; genotype)	Trainor et al. (2017)
Metabolomics	**DNN**, RF, SVM, DT, LDA, NSC, GBM	162 metabolites	271 profiles	Binary Classification (Diagnosis; breast cancer stratification)	Alakwaa et al. (2018)
Metabolomics	SVM, PLS-DA	16 and 131 metabolites	21 and 32 profiles	Binary Classification (Diagnosis; gestational diabetes mellitus)	Leitner et al. (2017)
Proteomics	LDA, SVM, kNN, RF	123 peptides	183 profiles	Multi-class Classification (Physiology; genotypes)	Hoehenwarter et al. (2011)
Transcriptomics	**CNN**, RF, DT, AdaBoost	60483 genes	6216 profiles	Multi-class Classification (Diagnosis; different cancer types)	Sharma et al. (2019)
Transcriptomics	SimNet	≤17814^b^ genes	348 profiles	Binary Classification (Diagnosis; breast cancer stratification)	Pai et al. (2019)
Transcriptomics	SimNet	not assigned^a^	194 profiles	Binary Classification (Diagnosis; asthma)	Pai et al. (2019)
Transcriptomics	SVR, RF, DNN, BEMKL, BRF, MMANN	≥68^c^ genes	1229 profiles	Regression (Physiology; eukaryotic growth rate)	Culley et al. (2020)
single-cell Transcriptomics	GNN	862 genes	162 single-cell profiles	Multi-class Classification (Other; cell type)^d^	Alghamdi et al. (2021)
single-cell transcriptomics	**CapsNet**, SVM, RF, LDA, kNN, ANN	3346 genes	17933^a^ single-cell profiles	Multi-class Classification (Other; cell type)^d^	Wang et al. (2020)
single-cell transcriptomics	CapsNet	9437 genes	4993 profiles	Multi-class Classification (Other; cell type)^d^	Wang et al. (2021)
Epigenomics	**VAE in combination with different ML methods**, RBF SVM, RF, ANN, DNN	438831 DNA methylation sites	3905 profiles	Multi-class Classification (Diagnosis; brain cancer subtypes)	Zhang et al. (2021)
Multi-omics (DNA copy number, Transcriptomics, Proteomics)	**modified NSC**, SVM, NSC	≤16266^a^ proteins, ≤17282^a^ genes	103 profiles per omics-type	Multi-class Classification (Diagnosis; breast cancer stratification)	Koh et al. (2019)
Multi-omics (Transcriptomics, Proteomics, microRNA Transcriptomics, DNA methylation, DNA copy number)	SimNet	not assigned^a^	150, 252, 77 and 155 profiles per omics-type in four independent data sets	Binary Classification (Clinical Outcome; cancer patient survival)	Pai et al. (2019)
Multi-omics (mRNA Transcriptomics, microRNA Transcriptomics, Epigenomics)	**VAE in combination with different ML methods**, RBF SVM(R), RF(R), ANN(R), DNN(R), CoxPH	58043 genes, 438831 DNA methylation sites, 1881 miRNAs	9736–11538 profiles per omics-type	Multi-class Classification (Diagnosis; different cancer types), Regression (Physiology; patient age), Binary Classification (Physiology; patient biological sex), Multi-class Classification (Physiology; tumour stage, Other; body region of tumor emergence), Regression (Clinical Outcome; patient survival function)	Zhang et al. (2021)
Multi-omics (Proteomics, Metabolomics)	SVM, GLM, NSC, RF, LDA, DNN	≤141^b^ metabolites, ≤ 27^a^ proteins	26 profiles per omics-type	Binary Classification (Clinical Outcome; perinatal outcome in asymptomatic women with short cervix)	Bahado-Singh et al. (2019)
Multi-omics (Transcriptomics, SNP-omics (genetic variants))	**DNN with Lasso**, DSPN, AdaBoost, DT, SVM, ANN, RF, kNN, GP, NBC, RBM, RBF SVM, SVM with Lasso, LogReg with Lasso	2598 genes, 127304 SNPs	1378 profiles per omics-type	Binary Classification (Diagnosis; schizophrenia)	Nguyen et al. (2021)
Multi-omics (Transcriptomics, SNP-omics (genetic variants))	**DNN with Lasso**, DSPN, AdaBoost, DT, SVM, ANN, RF, kNN, GP, NBC, RBM, RBF SVM, SVM with Lasso, LogReg with Lasso	118 genes, 332 SNPs	248 profiles per omics-type	Binary Classification (Diagnosis; lung cancer stage)	Nguyen et al. (2021)
Multi-omics (Transcriptomics, Proteomics, Metabolomics, Fluxomics)	RNN, LassoReg, Ensemble LassoReg	4096 genes, 1001 proteins, 356 metabolites, ≤120 ^b^ fluxes	≤3579^b^ transcriptomics profiles, ≤71^b^ proteomics profiles, ≤696^b^ metabolomics profiles, ≤43^b^ fluxomics profiles	Regression (Physiology; expression level of mRNAs, proteins and metabolites, prokaryotic growth rate)	Kim et al. (2016)
Multi-omics (Fluxomics, Metabolomics)	DT	≤106^a^ metabolites, ≤175^a^ fluxes	not assigned^a^	Binary Classification (Other; feasibility of kinetic parameter sets)	Andreozzi et al. (2016)
Multi-omics (time-series Proteomics and Metabolomics)	Models found by TPOT	≤86^e^ metabolites,≤76^e^ proteins	21 profiles per omics-type	Regression (Physiology; metabolite time derivatives)	Costello and Martin (2018)

a True number not clearly obvious from the descriptions found in the main body of the work.

b Number might be lower because some (additional) raw features or samples might have been filtered out.

c Value varies between different prediction methods.

d This prediction task was repeated on other data sets from the same omics type(s) that are not listed here.

e Estimated from provided supplementary material.

*Table notes:* Counts for effective raw features/samples are explained in detail in Section 2.1. Additionally, non-omics features are not listed. The listed prediction methods are generic types, meaning that they may describe any derived method. Please consult the referenced publications for details on the utilized method. Supervised methods that were not used for predictions but e.g., in preprocessing, the post-hoc phase, or for additional analysis are not listed. Bold methods indicate which methods were presented as the authors’ methods of choice or which were primarily used for predictions. Abbreviations: DNN, Deep Neural Network; RF, Random Forest; SVM, Support Vector Machine; DT, Decision Tree; LDA, Linear Discriminant Analysis; NSC, Nearest Shrunken Centroid; GBM, Gradient Boosting Machine (Boosted Tree Model, Generalized Boosted Model, Gradient Boosted Tree); TPOT, Python package for automatic model selection (see Supplementary Table S1); PLS-DA, Partial Least Squares Discriminant Analysis; RBF, Radial Basis Function Kernel; ANN, feed-forward Artificial Neural Network; LogReg, Logistic Regression; XGBoost, Extreme Gradient Boosting; Lasso, Lasso (L1) Regularization; LassoReg, Lasso Regression; SVR, Support Vector Regression; BEMKL, Bayesian Efficient Multiple Kernel Learning; BRF, Bagged RF; MMANN, Multi-Modal ANN; VAE, Variational Autoencoder; RNN, Recurrent Neural Network; Ensemble *X*, combination of multiple base models of type *X*; GNN, Graph Neural Network; NBC, Naïve Bayes Classifier; CapsNet, Capsule Network; GLM, Generalized Linear Model; LGP, Linear Genetic Program; AdaBoost, Adaptive Boosting; GP, Gaussian Process; RBM, Restricted Boltzmann Machine; SimNet, Similarity Network; *X*(R), Regression variant of method *X*; CoxPH, Cox Proportional Hazard Model; miRNA, micro Ribonucleic Acid; SNP, Single-Nucleotide Polymorphism; kNN, k-Nearest Neighbors; CNN, Convolutional Neural Network; DSPN, Deep Structured Phenotype Network.

### 2.1 Data set dimension and size

The number of features (i.e., data set dimension) and samples (i.e., data set size) can be an important factor for a ML model’s performance. Alakwaa et al. (2018) found that their neural network model under-performed when data set size was low but out-performed other ML methods when the training set was sufficiently large. Further, Mendez et al. (2019) compared the performance of several ML models on multiple metabolomics data sets and suggested that, at least in their study, classification error was impacted less by a change in the ML method than by a change in the number of training samples. We have, therefore, also included this information in Table 1. However, one should be explicit when listing data set dimensions and sizes. In a ML project, the original data set is often heavily processed: original features are scaled, new features are created, some original samples or features are omitted, etc. In this work, we summarize the part of the workflow that starts after raw data tables have been constructed and manipulates data before it reaches the ML model for prediction as *data preprocessing*. A raw data table in this context summarizes one omics type and contains one value per omics entity for every observed entity (e.g., one abundance value per metabolite for every patient). Data preprocessing is outlined in more detail in Section 2.2. Preprocessing often changes the dimension and size of a data set, sometimes creating completely new features and samples. As an example, Toubiana et al. (2019) derived a set of 444 graph-based features for 339 pathways from a few repeated profiles of 106 metabolites by characterizing pathways in metabolite correlation networks. Sample conversions that change the entity a sample belongs to, e.g., from a “biological replicate” to a pathway (Toubiana et al., 2019), seem relatively rare. However, since feature conversions are frequently encountered (Andreozzi et al., 2016; Koh et al., 2019; Pai et al., 2019; Sharma et al., 2019; Toubiana et al., 2019; Culley et al., 2020; Zhang et al., 2021) we need to clarify what the numbers found in Table 1 mean.

Typically, specifications of dimension and size characterize only either the raw data set or the ML-ready data set used in optimizing and testing a ML model. In our opinion, a reasonable alternative approach to express data set dimensions and sizes is one that quantifies the amount of raw data that ultimately contributes to the ML-ready data set. We call the corresponding values *effective raw feature/sample counts*. These metrics describe the number of raw features (i.e., variables of genes, SNPs, DNA methylation sites, proteins, metabolites, fluxes, etc.) and raw samples (e.g., omics feature profiles) from the raw data sets that contribute information to a single data set available for ML. Hence raw features or samples that are not integrated into the ML-ready data set because they were filtered out during preprocessing are not counted towards these values. However, even if raw features partially become target variables (Kim et al., 2016) they can still be considered effective. Since effective raw features and samples are part of the raw data set, it is important to not confuse their counts with specifications that refer to final features and samples of the ML-ready data set, which might be quite different. We argue that effective raw feature and sample counts allow comparison of ML-ready data sets even under extreme data set transformations and reductions. Although these numbers seem relevant they are unfortunately often difficult to reconstruct from a reader’s perspective without analysing the original data and code. Further, when the same raw data set yields multiple distinct ML-ready data sets, effective counts can vary a lot between models, as noticeable in the study by Culley et al. (2020).

**FIGURE 1:**
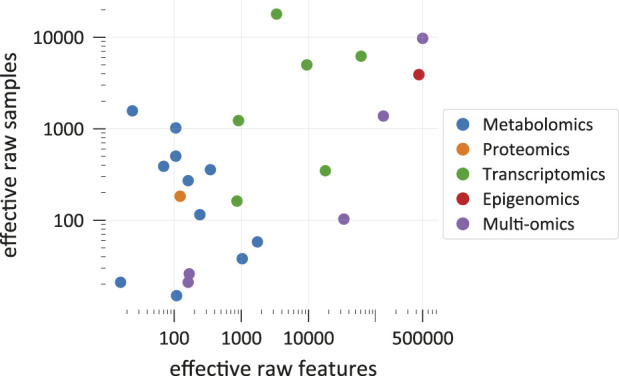
Comparison of effective raw data set dimensions and sizes in the categorized studies. Each point represents a data set that was used for optimizing and testing at least one predictive model. In *multi-omics*, a data set includes measurements from multiple omics sources. Each data set is plotted at the position that corresponds to its effective raw dimension and size. Please refer to the main text for explanations on the meaning of effective raw feature and sample counts (Section 2.1). Note that the graph shows only a selection of all ML-ready data sets from all studies. Supplementary Figure S1 provides references to the shown data points.

Figure 1 shows effective counts for ML-ready data sets in the 26 categorized publications. Generally, we find that studies that use solely metabolomics data (Leitner et al., 2017; Liu et al., 2017; Trainor et al., 2017; Alakwaa et al., 2018; Asakura et al., 2018; Date and Kikuchi, 2018; Hu et al., 2018; Stamate et al., 2019; Toubiana et al., 2019; Sha et al., 2021; van Dooijeweert et al., 2021) use a lower number of effective raw features for predictions than studies employing only transcriptomics (Sharma et al., 2019; Culley et al., 2020; Wang et al., 2020; Alghamdi et al., 2021; Wang et al., 2021). The two exceptions on the transcriptomics side (Culley et al., 2020; Alghamdi et al., 2021) originally had more raw features but some of them were omitted for at least one major analysis because some genes were not present in a metabolic network model. Due to technical limitations, metabolomics still struggles to reach high throughputs, such that either the number of raw features or the number of raw samples is restricted. This depends also on the experimental method. All metabolomics studies in Figure 1 with more than 200 effective raw features (Trainor et al., 2017; Stamate et al., 2019; Sha et al., 2021) use liquid chromatography coupled to mass spectrometry (LC-MS) or LC-MS together with another method, respectively. While the study with the second-lowest number of effective raw features (Liu et al., 2017) used LC-MS together with nuclear magnetic resonance (NMR) spectrometry, in this case, the authors reduced their raw feature count from originally 261 to 24 effective metabolite features for predictions. Although methods of 2-dimensional gas chromatography can detect respectable amounts of molecules (Phillips et al., 2013), studies that used solely gas chromatography (Alakwaa et al., 2018) or NMR (Asakura et al., 2018; Date and Kikuchi, 2018) did not reach more than 200 compounds. Another concern of metabolomics is that the exact identity of some of the raw features is often unclear (Weckwerth, 2011). Recently, some efforts have been made to solve this metabolite annotation problem also with machine learning approaches (Nguyen et al., 2019). The biological meaning of features is especially important when results should be interpreted. Consequently, interpretation methods that evaluate the importance of individual features might struggle to generate meaningful biological insight when applied to metabolomics data with unreliable annotations.

On the other end of the scope, transcriptomics oftentimes easily reaches over 3,000 effective raw features (Sharma et al., 2019; Wang et al., 2020; Wang et al., 2021) and studies that use measurements from multiple omics sources can have and retain close to 500,000 raw features due to the high-dimensionality of epigenomics data and strategies to condense this information (Zhang et al., 2021). However, taking into account more features for a prediction is not always favourable. Besides technical difficulties linked to data sets with many features, like storing large feature vectors and computational cost (Bommert et al., 2022), working with high-dimensional samples causes diverse issues. The machine learning literature summarizes challenges that arise in high-dimensional data sets under the “curse of dimensionality” (Bishop, 2006; Shalev-Shwartz and Ben-David, 2013; Forsyth, 2019). Especially, when relevant information in the data is “sparse,” meaning that only a few features truly influence the prediction target, like it is often the case for transcriptomics data (Vikalo et al., 2007), considering additional features only “add[s] noise to the data” (Culley et al., 2020). Having high-dimensional samples, while the number of samples is much lower, is even worse. One major problem is that the same number of samples are often spread over wider distances in a higher-dimensional space (Forsyth, 2019, p. 77f) and it would, therefore, require much more samples to similarly populate this space (Bishop, 2006, p. 35). In this case, the risk of overfitting to the training data is increased (Kim and Tagkopoulos, 2018; Jiang et al., 2020). A way to mitigate the “curse” is by reducing the number of dimensions by combining original features to find a new lower-dimensional description for each original sample or by omitting some original features (Zhang et al., 2021). The corresponding methods are often called *feature extraction* and *feature selection* and summarized as *dimensionality reduction techniques* (Reel et al., 2021). These methods are frequently “unsupervised,” meaning that they do not use the information stored in the labels (Cai et al., 2022) and are almost always advisable when dealing with a large number of raw features. Feature selection methods can make ML models more accurate (Chen et al., 2020) and better interpretable (Bommert et al., 2022). For more details, see the following section about data preprocessing (Section 2.2).

In addition, sometimes omics data such as metabolite amounts reference information that is changing over time. These dynamics are important to consider when modeling with data collected at multiple time points, as it may affect the reliability of ML predictions. One possible innovation for correcting algorithms that have to deal with input data representing dynamic information is by analysing *concept drift* (Agrahari and Singh, 2021). Concept drift in machine learning arises when the statistical properties of the target variable change over time, usually due to the fact that the identity of the input data that the model was trained on has significantly changed over time. Then, a model that is unaware of this change can no longer make accurate predictions. It has already been shown that metabolomics data is subject to concept drift, making prediction models not taking the dynamics into account less reliable (Schwarzerova et al., 2021).

### 2.2 Data preprocessing

**FIGURE 2:**
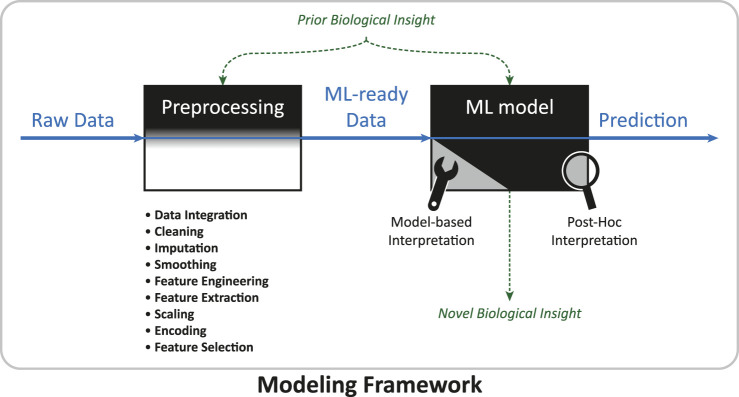
Data and information flow in a modeling framework. The modeling framework inhabits the complete work-flow of a machine learning project, from the raw data set to producing a final prediction. Data preprocessing converts the raw data set into a data set suitable for machine learning. In the machine learning phase, model-based or post-hoc interpretation methods might be applied to generate novel biological knowledge. Prior biological insight (see Table 2 for examples) might enter at different steps, sometimes improving the interpretability of the ML model.

In the machine learning community there is a popular saying: “garbage in, garbage out.” It means that every successful machine learning project lives and dies with the quality of the data set it uses. Besides the experimental procedure that determines the raw data quality, data preprocessing, the step that takes raw data and turns it into a data set suitable for learning, is critical (Kotsiantis et al., 2007), especially for omics data (Kim and Tagkopoulos, 2018). Figure 2 illustrates the flow of data and information through a modeling framework, indicating the vital role of data preprocessing. Data preprocessing can involve many steps, and these often heavily depend on the raw data and application. In particular, during preprocessing

• data from different sources might be combined (**data integration**), e.g., microRNA and mRNA expression levels might be “concatenated” (Cai et al., 2022),
• samples might be deleted (**cleaning**), e.g., because a patient might be an obvious outlier, the diagnosis is unclear, or a value is obviously corrupted like a negative abundance record,
• missing values need to be filled in (**imputation**), e.g., by inferring them from other measurements,
• noise might be reduced (**smoothing**), e.g., by “smoothing methods” (Simonoff, 1996),
• new features and data representations might be created with the help of dimensionality reduction techniques and/or expert knowledge (**feature extraction** [Guyon and Elisseeff, 2006] and **feature engineering** [Kuhn and Johnson, 2019]), e.g., an “autoencoder” (see Section 3.3.2 for explanation) might find a compact vector description of a large epigenomics profile (Zhang et al., 2021), or the fluxome might be inferred from transcript levels via constraint-based models (Culley et al., 2020),
• the scale of variables might be changed (**scaling**), e.g., normalizing and/or standardizing gene expression values within genes,
• the format of variables might be changed (**encoding**), e.g., “0” might indicate absence of a gene and “1” its presence (Kim et al., 2016),
• a subset of the initial variables might be selected (**feature selection**), e.g., some metabolite features can be disregarded because they are linked to pharmacotherapy of the disease of interest (Liu et al., 2017) or because they were previously reported to be irrelevant for disease prediction.

There is no universal recipe that, when applied to any data set, will yield good results (Kotsiantis et al., 2007). Hence, finding a preprocessing procedure that works well for a given problem sometimes requires testing several methods (Forsyth, 2019, p. 376). In many cases some preprocessing steps are not needed or they might need to be done in a different order. Additionally, prior biological knowledge might enter into the modeling framework at several points throughout preprocessing. A few examples are as follows: Culley et al. (2020) incorporated a genome-scale metabolic model into their modeling framework to derive simulated fluxome-level features by bounding reactions with experimental transcriptomics data. Pai et al. (2019) created features for groups of genes from transcript-level features by using known gene-pathway associations. Andreozzi et al. (2016) used prior knowledge about the kinetic properties of enzymes to help create multiple kinetic models that served as input to their machine learning model. Koh et al. (2019) used biological networks to calculate interaction-level features from the abundances of interaction partners (i.e., genes and proteins). Possibilities in finding new data representations seem very diverse. Omics profiles can be converted to images by mapping expression levels of genes or pathways onto pixels with unsupervised techniques, making them accessible for “convolutional neural networks” (Sharma et al., 2019; Oh et al., 2021), which are explained later in Section 3.3. Autoencoders can condense almost 500,000 biological features from three omics sources into a single feature vector with 128 entries informative for several subsequent predictions (Zhang et al., 2021).

Although preprocessing can reduce computational cost and significantly improve predictions (Zhang et al., 2019), it can also hurt performance when valuable information is accidentally thrown away during a preparation step (Bishop, 2006, p. 3; Guyon and Elisseeff, 2006, p. 4). This is observable in the work of Culley et al. (2020). In their performance comparison, distinct regression models that were trained on original experimental transcriptomic features consistently out-performed those trained on artificial flux features derived from the same experimental data. Culley et al. (2020) observed only performances similar to ML models trained solely on the original data when they combined information from the original and converted data. In one case, the integrated data slightly outperformed the original gene expression data. This example may demonstrate that mechanistic insight (e.g., constraint-based modeling) can enrich experimental data (Culley et al., 2020). Nonetheless, converting features from one omics layer to another should be done with care, since blindly trusting new features while disregarding the original data could lead to poorer results (Guyon and Elisseeff, 2006, p. 4). For details on how to prepare raw omics data sets for machine learning the work of Kim and Tagkopoulos (2018) is a good starting point. Further, there are great books (Guyon and Elisseeff, 2006; Kuhn and Johnson, 2019) for learning how to manipulate and select features in order to improve performance.

A common problem in omics data sets is that the number of features is much higher than the number of samples. In that case, dimensionality reduction through feature extraction, engineering, or selection is useful to reduce the impact of data sparsity on the prediction reliability.

## 3 Toolbox for supervised machine learning

With the growing interest in machine learning in recent years, the toolbox of available methods and platforms to apply them grows constantly. As a consequence, selecting a method that works well for a given task and data set can be daunting for non-experts in the field of data science. There is “no free lunch” (Wolpert and Macready, 1997) in supervised machine learning, meaning that there exists no “universal” model that works well in any situation (Shalev-Shwartz and Ben-David, 2013, sect. 5.1). Instead expertise about the specific biological problem is important for a successful ML project (Shalev-Shwartz and Ben-David, 2013, sect. 5.1.1). In this section, we provide an overview of some of the supervised learning methods that have been applied to omics data sets. Due to the sheer diversity of methods that have been introduced to systems biological problems (see Table 1), describing them all in detail would go beyond the scope of this work.

From a very general point of view, supervised learning is the task of learning a mapping (a “hypothesis”; Shalev-Shwartz and Ben-David, 2013, sect. 2.1) between a set of variables (the features) and one or more target variables (the labels) given a set of pairs of these two (the training data) to discriminate among target variables (Angermueller et al., 2016). The ML model normally receives features in the form of a vector (Angermueller et al., 2016). By convention this feature vector is denoted $x\in R^{d}$ , where *d* is the dimension of the vector (Bishop, 2006; Forsyth, 2019; Deisenroth et al., 2020). For simplicity, we will now consider only the case where there is a single target variable. Depending on the type of this label one discriminates between two categories of supervised machine learning methods, namely *classification* and *regression*. In a classification problem setting, a label, *y*_*i*_, describes to which class a sample, *i*, belongs and can take one of two in binary classification (*y*_*i*_ ∈ {*C*_0_, *C*_1_}) or one of many possible values in multi-class classification (*y*_*i*_ ∈ {*C*_0_, *C*_1_, *…* , *C*_*n*_}). If our goal is to predict if a tumor belongs to a cancer subtype, possible classes could be: “subtype-A,” “subtype-B,” or “subtype-C,” which could be encoded to the numerical values {0, 1, 2}. For regression the label is a real number, $y_{i}\in R$ (Deisenroth et al., 2020, p. 289).

When using a training set of the form *T* = {(**x**_0_, *y*_0_), (**x**_1_, *y*_1_), *…*, (**x**_*N*_, *y*_*N*_)} (Deisenroth et al., 2020, p. 370) the task for a supervised ML algorithm is now to select a suitable hypothesis (Shalev-Shwartz and Ben-David, 2013, chpt. 2). A hypothesis maps a feature vector, **x** ∈ *X*, to a label, *y* ∈ *Y*, *h*: *X* → *Y* (Shalev-Shwartz and Ben-David, 2013, sect. 2.1). During learning, the algorithm picks from a set of possible hypotheses, the *hypothesis class*, h∈H (Shalev-Shwartz and Ben-David, 2013, sect. 2.3). To tell the learning algorithm which hypothesis works well, we have to define a criterion that measures how large the error is between the true label (*y*_*i*_; known from the training data) and a prediction made by the model, ${\hat{y}}_{i}=h\left(x\right)$ . This criterion is known as a *loss function*, $l_{h}\left(y_{i},{\hat{y}}_{i}\right)$ (Deisenroth et al., 2020, p. 260). The optimization problem is now to minimize the mean error over all our training samples (Deisenroth et al., 2020, p. 260; Shalev-Shwartz and Ben-David, 2013, sect. 2.2 and 2.3). This is known in statistical learning theory as *empirical risk minimization (ERM) with inductive bias* (Shalev-Shwartz and Ben-David, 2013, sect. 2.3).

It is important to note that we usually want to find a model that minimizes the error on data not presented during training (Deisenroth et al., 2020, p. 261), like samples from patients we want to diagnose in order to give them the right medical treatment. However, minimizing this error would require unlimited training samples (Deisenroth et al., 2020, p. 261). The fact that we have only access to a restricted training set (Deisenroth et al., 2020, p. 262) is why one should always test a trained model on data the model was not fit to. A predictor that performs well on training data but poorly on new data has learned a bad hypothesis, one that does not generalize to new samples drawn from the same data-generating distribution (Maceachern and Forkert, 2021), as mentioned earlier in Section 2.

### 3.1 Classification

#### 3.1.1 Support vector machine

Support Vector Machines (SVM) are frequently used for binary classification purposes (Leitner et al., 2017; Alakwaa et al., 2018; Date and Kikuchi, 2018; Sha et al., 2021; van Dooijeweert et al., 2021). In this basic setting, SVMs aim to find a “decision boundary” in the form of a hyperplane (Bishop, 2006, p. 326f) that segregates the two classes of data points (Forsyth, 2019, p. 21). In the case where the two classes are perfectly separable there exists an endless number of possible hyperplanes that correctly classify all training samples (Deisenroth et al., 2020, p. 374). SVMs select the hyperplane that lies half-way between the two data point clusters. More specifically, they choose the hyperplane that is farthest away (in terms of “perpendicular distance”) from the nearest data point (Bishop, 2006, p. 327). SVMs can also be applied to problems where classes are not perfectly separable (Cortes et al., 1995) by permitting some data points to be incorrectly labeled (Deisenroth et al., 2020, p. 379). The error function that allows SVMs to find an optimal solution is the *hinge loss* (Forsyth, 2019, p. 23). There are several great books that introduce SVMs in detail (Bishop, 2006; Shalev-Shwartz and Ben-David, 2013; Forsyth, 2019; Deisenroth et al., 2020).

Support vector machines are probably one of the most classical machine learning methods and frequently serve as base-line models in performance comparisons for omics data sets (Asakura et al., 2018; Date and Kikuchi, 2018; Koh et al., 2019; Wang et al., 2020; Nguyen et al., 2021; Sha et al., 2021). van Dooijeweert et al. (2021) chose a SVM as their primary method to classify individuals based on their metabolomics signatures as either healthy or potentially having Diamond Blackfan Anaemia (DBA).

#### 3.1.2 Decision trees, random forests and boosted trees

Decision trees classify samples based on a tree-like hierarchical decision process. Starting from a *root node* and proceeding towards one of many *leaf nodes*, a sample is classified by following a path within the tree that is controlled by making a decision at each step (i.e., at each “internal node”; Shalev-Shwartz and Ben-David, 2013, chpt. 18). A final decision leads to a leaf that determines the class label for the given sample (Shalev-Shwartz and Ben-David, 2013, chpt. 18). Decisions within the tree use certain properties of the sample, which can be viewed as asking a yes/no question similar to, Is the expression of gene A higher than a threshold? and then proceeding along the corresponding branch (Shalev-Shwartz and Ben-David, 2013, chpt. 18). Decision trees can be automatically constructed by repeatedly choosing questions (“splitting rules”) from a pool of questions while each time evaluating the benefit of using a particular question with the help of a *gain measure* (Shalev-Shwartz and Ben-David, 2013, sect. 18.2).

The ability to verbalize and visualize a decision tree in terms of simple yes/no questions makes them a common example of a likely interpretable machine learning method (Shalev-Shwartz and Ben-David, 2013; Lipton, 2016; Murdoch et al., 2019). As long as its “depth” [i.e., the number of decisions to reach a leaf (Shalev-Shwartz and Ben-David, 2013, sect. 21.1)] stays within the limits of human comprehension a decision tree is usually a simulatable classifier (see Section 4.1 for explanation) as implied by Lipton (2016). However, decision trees have a known disadvantage, i.e., a single decision tree of arbitrary size tends to overfit data (Shalev-Shwartz and Ben-David, 2013, sect. 18.1 and 18.2). By combining multiple decision trees into a *random forest* (Breiman, 2001), letting them “vote” on labels, and choosing the one that gets the most votes, overfitting can be circumvented (Shalev-Shwartz and Ben-David, 2013, sect. 18.3). Using a “**b**ootstrap **agg**regat**ing**” (short “**bagging**”) method (Breiman, 1996) is a common way to construct random forests (Forsyth, 2019, p. 41f).

Another approach that combines decision trees is *boosting* (Friedman, 2002). In short, boosting constructs a series of “base” models (e.g., decision trees) in which each model has a different voting power and they are trained such that more attention is brought to samples incorrectly labeled by earlier models (Bishop, 2006, p. 657). For a more detailed description of random forests and boosting please refer to the work of Breiman (2001) or to Bishop’s (2006) book for bagging and boosting. Andreozzi et al. (2016) provide an illustrative toy example of a decision tree and demonstrate how the rules learned by the tree can be utilized to improve the “feasibility” of a population of kinetic models. Similar to support vector machines, random forests are popular for performance comparisons in systems biology (Alakwaa et al., 2018; Asakura et al., 2018; Date and Kikuchi, 2018; Wang et al., 2020; Nguyen et al., 2021; Sha et al., 2021).

#### 3.1.3 k-nearest neighbors

k-nearest neighbors (kNN) is a method that classifies new data points based on how similar they are in their features to samples in the training data set for which the true class label is known (Forsyth, 2019, p. 7). More specifically, a new sample is given the label that is most probable when looking at its *k* *-nearest neighbors* (Bishop, 2006, p. 125f) in terms of an appropriate measure of distance in feature space (Forsyth, 2019, p. 8). kNN classifiers are sometimes used in performance comparisons (Trainor et al., 2017; Wang et al., 2020; Nguyen et al., 2021), however, from the 26 considered studies in this review, none presented kNN as their method of choice for predictions.

#### 3.1.4 Nearest shrunken centroid

Nearest shrunken centroid (NSC) is a modified version of the nearest-centroid classifier and was proposed by Tibshirani et al. (2002) for inferring tumor classes from trancriptomics data. Its advantage over the original classifier (i.e., nearest-centroid) lies in that it allows for an inherent selection of features that are most distinct between sample classes (Tibshirani et al., 2002). Thus, it is suitable for data sets with a high number of features that may simultaneously contain only a few relevant signals like transcriptomics data.

Following the steps in the original publication (Tibshirani et al., 2002): First, the algorithm calculates an average sample (i.e., the *centroid*) for each class and the whole data set. Then, the similarity between the class centroids and the global centroid is evaluated by a *t*-statistic for every feature and class. This *t*-statistic is then numerically “shrunken” by subtracting a constant, Δ. In the final classifier, a feature effectively loses its ability to distinguish between classes if all of its corresponding values dropped beneath zero or became zero in this step. This way, features that are unimportant for predictions can be gradually removed as Δ increases (Tibshirani et al., 2002). Koh et al. (2019) modified the original version of NSC such that it takes into account also related features when calculating test statistics.

### 3.2 Regression

Regression is the task of finding a mapping from a feature vector to a real number (Jiang et al., 2020). In a regression setting, a fundamental assumption is that our labels are subject to some random measurement error; hence, there is no relationship between the labels and features in the form of a deterministic function (Deisenroth et al., 2020, p. 289). An example of a regression problem would be the prediction of an organism’s body size from metabolomic measurements (Asakura et al., 2018).

#### 3.2.1 Linear regression

In *linear regression* we assume that a straight line that is randomly displaced from the origin relates features and labels (Forsyth, 2019, p. 209). Given a training data set, suitable model parameters (a.k.a. fitting the line) are usually found by so-called “maximum likelihood estimation” using a “gradient descent” algorithm (Deisenroth et al., 2020, p. 293), which is, in this context, the same as finding the minimum of the sum of squared residuals between model predictions and the training labels (Bishop, 2006, p. 141).

#### 3.2.2 Lasso regression

Lasso is a *regularization method* that was proposed by Tibshirani (1996) and can eliminate non-informative features by setting their contributions to zero, potentially yielding a *sparse model* (i.e., a model that effectively uses only some of the given features; Forsyth, 2019, p. 262f). Generally, regularization tries to avoid overfitting during training, e.g., by keeping parameters in reasonable ranges, embedding feature selection into the model (Jiang et al., 2020), or randomly switching neurons on and off in a neural network (Angermueller et al., 2016). In lasso regression, this is achieved by adding a regularization term to the loss function of the regression model that shrinks some parameters to zero, eliminating the contributions made by the corresponding features (Bishop, 2006, p. 144f). Kim et al. (2016) primarily used lasso regression in their modular ML approach to predict quantities in several omics layers and Nguyen et al. (2021) incorporated lasso regularization into their deep neural network for selecting predictive features. Lasso regression was also applied to omics data as a feature selection strategy for the final predictive model (Leitner et al., 2017; Liu et al., 2017; Pai et al., 2019). Leitner et al. (2017) used this approach to select for the most suitable set of metabolites for early prediction of gestational diabetes mellitus (GDM). A combination of two different data sets, blood and urine samples, showed the highest prediction accuracy with a SVM model.

#### 3.2.3 Partial least squares regression

Partial least squares (PLS) regression was introduced by Wold (1975) and constructs a set of *latent variables* that are most predictive of multiple target variables from the original features (Abdi, 2010). PLS works well when there are less samples than features and when features are suspected to be highly correlated with each other (Abdi, 2010; Trainor et al., 2017). Consequently, metabolomics data lends itself to PLS, e.g., because of its oftentimes low number of samples with many features and correlated metabolites (Mendez et al., 2019). Additionally, PLS is well-accessible for post-hoc interpretations that measure feature importance (Fonville et al., 2010; Leitner et al., 2017; Mendez et al., 2019).

A variant of PLS that is sometimes used to classify omics profiles is *partial least squares discriminant analysis (PLS-DA)* (Trainor et al., 2017; Date and Kikuchi, 2018). In this case, the target variables are categorical and a threshold on the predictions made by a corresponding regression model determines the predicted labels (Brereton and Lloyd, 2014).

For in-depth mathematical descriptions of the regression and the classification approach, see Abdi (2010) and Brereton and Lloyd (2014). Fonville et al. (2010) discuss some interpretability aspects of PLS and related methods in metabonomics.

### 3.3 Neural networks

Neural networks comprise a large group of machine learning methods that all have in common that they contain entities called *neurons* (Sengupta et al., 2020). Real biological neurons and how they wire and learn together initially served as a model for these mathematical units (Macukow et al., 2016). Nonetheless, modern artificial neural networks (ANN) have only little in common with nervous systems. A neuron can be seen as a function that takes an input feature vector, **x**, and returns a value, *y*, that represents its current *activity* (Angermueller et al., 2016). A typically non-linear *activation function* determines how the neuron responds to inputs weighted by learnable *weight* parameters (Mendez et al., 2019; Sengupta et al., 2020). Another learnable parameter, the *bias*, is added before the input-to-output conversion and determines how easily the neuron activates (Sengupta et al., 2020). Generally, one could speak of a neural network when a neuron receives input from another neuron.

In the most classical type of neural networks, called “feed-forward neural networks,” neurons are organized into *layers* (Mendez et al., 2019). Each layer holds a number of neurons that solely receive input from neurons in the previous layer and pass their output only to neurons in the next layer. However, some neurons might receive no input and instead show a steady activation (Shalev-Shwartz and Ben-David, 2013, sect. 20.1). Nonetheless, normally two consecutive layers are “fully connected,” meaning that every neuron in a subsequent layer receives a vector, **y** ^(*i*)^, corresponding to all outputs from a preceding layer (Angermueller et al., 2016). In a feed-forward neural network there are three types of layers. The *input layer* feeds the feature vector of a sample for which a prediction is to be made into the network. This input signal is then propagated through one or more *hidden layers* until the last layer, the *output layer*, is reached. The outputs, **y** ^(out)^, of the neurons in the output layer can for instance represent probabilities for cancer classes (Alakwaa et al., 2018) or even metabolite concentration change over time (Costello and Martin, 2018). In a binary classification task, the output layer often has only one neuron. At any hidden layer, an output vector, **y** ^(h)^, can be seen as a new set of internal “features” for an input sample abstracted automatically by the hidden neurons from their input vector (LeCun et al., 2015). This ability, to sequentially find new, more discriminative, features, allows feed-forward neural networks to enrich the information relevant for predictions (Forsyth, 2019, p. 367) and filter out less relevant information (LeCun et al., 2015).

When neural networks contain more than one hidden layer they are often termed “multilayer” or *deep neural networks (DNNs)* (Shrestha and Mahmood, 2019; Zhang et al., 2019). Deep neural networks have the advantage that they avoid having to carefully construct (i.e., “hand-engineer”) input features—instead the original raw features can be used directly in most cases (LeCun et al., 2015). *Backpropagation* is the key ingredient that allows DNNs to learn efficiently (Macukow et al., 2016). During backpropagation, the model’s prediction error is traced back to individual model parameters, hence allowing them to be appropriately adjusted (LeCun et al., 2015).

Neural networks can be applied to a variety of problems (Shrestha and Mahmood, 2019). When we allow neural networks with a particular activation function to have an unlimited number of hidden layers they can theoretically simulate any function connecting input features and target variables (Hanin, 2019).

#### 3.3.1 Specialized neural networks

There are a lot of different neural network architectures that were mostly designed to perform well on one specific task. Examples of specialized neural networks that have been applied to omics data sets are *convolutional neural networks* (Sharma et al., 2019; Oh et al., 2021), *recurrent neural networks* (Kim et al., 2016), *graph neural networks* (Alghamdi et al., 2021), *capsule networks* (Wang et al., 2020; Wang et al., 2021), and *autoencoders* (Zhang et al., 2021).

Convolutional neural networks (CNNs) were developed to work with data in which features have a known spatial relation, e.g., sequential data, image-like data, and stacks of image-like data (LeCun et al., 2015). They can learn to recognize complex objects such as animals in pictures by internally decomposing their input (LeCun et al., 2015). This ability is partly due to the fact that consecutive layers are not fully linked such that a neuron sees only a part of the whole picture, the “local receptive field” (Shrestha and Mahmood, 2019). Sharma et al. (2019) applied CNNs to transcriptomics data by assigning RNAs to pixels according to their similarity in the training data and then integrating RNA abundances into these pixels for every sample.

Recurrent neural networks (RNNs) perform well on time-series data, where “information of previous time steps” needs to be remembered because it is relevant for later time points (Sengupta et al., 2020). Unlike in classical feed-forward architectures (e.g., multi-layer feed-forward neural networks), in RNNs, neurons receive information extracted from earlier inputs additionally to the present input (Sengupta et al., 2020). Kim et al. (2016) used a RNN to predict transcript levels in a cell from genetic and environmental features in the hope of replicating the behaviour of cycles frequently found in transcriptional regulatory networks.

Graph neural networks (GNNs) is an umbrella term for neural networks which can work with data that can be represented as graphs (Zhou et al., 2018) and there are many subtypes of them (Wu et al., 2019). For instance, “Message Passing Neural Networks (MPNN)” (Gilmer et al., 2017) are a type of “convolutional graph neural networks” (Wu et al., 2019) in which vertices in the graph store information and share information along edges with neighboring vertices in a step-wise process until an output is generated by taking into account the final states of vertices (Gilmer et al., 2017) for local “node-level” or global “graph-level” predictions (Wu et al., 2019). Alghamdi et al. (2021) used a GNN to infer metabolic reaction rates in individual cells from transcriptomics data by viewing the metabolic network as a factor graph.

In the next sections, we will discuss autoencoders and capsule networks in more detail. We highlight autoencoders because of their ability to serve as powerful feature extractors, as demonstrated on multi-omics data (Zhang et al., 2021), and capsule networks because of their young age and distinct nature to “regular” neural networks. Shrestha and Mahmood (2019) and Sengupta et al. (2020) review many more specialized neural network architectures, and Zhou et al. (2018) and Wu et al. (2019) discuss graph neural networks in great detail.

#### 3.3.2 Autoencoders

An autoencoder is a special feed-forward neural network architecture that, rather than trying to predict target variables from an input, learns to output its given input (Martorell-Marugán et al., 2019). Since they only use feature information they can be classified as “unsupervised DNN[s]” (Shrestha and Mahmood, 2019). The important detail about this architecture is that it includes a hidden layer with usually only a few neurons (Sengupta et al., 2020). This characteristic layer is sometimes called the *bottleneck*. Since information is passed on from layer to layer, at the bottleneck the model is forced to find a description of the input with low dimension (Sengupta et al., 2020). In contrast to principal component analysis for dimensionality reduction, non-linear activation functions allow autoencoders to compress their inputs non-linearly (Shrestha and Mahmood, 2019), which can lead to more informative descriptions (Charte et al., 2018). The bottleneck divides autoencoders into two parts, the *encoder*, and the *decoder* (Shrestha and Mahmood, 2019). While the encoder tries to extract the most relevant information from the original input to condense it at the bottleneck, the decoder tries to reproduce the input in the output layer from it (Shrestha and Mahmood, 2019). Once an autoencoder was trained, it can generate a compact description from a sample which may then serve as input for predictive models or can be used to plot the data when the new description has only two or three dimensions (Zhang et al., 2021).

There is a wide variety of autoencoders that can serve other purposes than just dimensionality reduction. For instance, when an autoencoder is challenged to reproduce original samples from samples that were randomly perturbed the model can learn to remove similar “noise” from new samples (Gondara, 2016). Another commonly used version is a *variational autoencoder (VAE)*. Rather than learning discrete sample descriptions, VAEs learn the parameters of a normal distribution from which new descriptions can be drawn (Zhang et al., 2021). As such an VAE can act as a sample generator that could theoretically come up with omics measurements for imaginary patients when decoding a newly drawn description (Shrestha and Mahmood, 2019; Zhang et al., 2021). Furthermore, model parameters learned by an autoencoder can serve as first drafts for those of a supervised neural network, allowing effective “pre-training” of supervised models (Erhan et al., 2010) as demonstrated on omics data (Alakwaa et al., 2018).

#### 3.3.3 Capsule networks

Capsule Networks (CapsNets) are a novel type of neural network that was introduced by the team of Geoffrey E. Hinton (Sabour et al, 2017). CapsNets have challenged the state-of-the-art CNNs in image identification. CapsNets aim to overcome some of the flaws of CNNs, like the loss of local information during a typical filter operation and difficulties with recognizing objects when they appear in new orientations (Sabour et al, 2017). CapsNets are exceptionally good at resolving objects when they are shown on top of each other (Sabour et al, 2017). According to the authors (Sabour et al, 2017), in a capsule network multiple neurons are configured into “capsules” that each detect the presence and characteristics of an associated “entity.” In an transciptomics profile, an individual capsule can be set up to predict the presence of a specific protein and indicate its properties (Wang et al., 2021). A capsule returns a vector that corresponds to the activities of its neurons and indicates the probability that the entity is present with its scale and the entity’s characteristics by its orientation (Sabour et al, 2017). Capsules are further organized into layers that follow a child-parent like hierarchy. As an example, in the implementation of Wang et al. (2020), the capsules in the last capsule layer each indicated the presence of a cell class that the authors aimed to predict. In a later work (Wang et al., 2021), child capsules of these parent capsules representing cell classes were encouraged to portray transcription factors or groups of interacting proteins. When processing samples, an innovative *dynamic routing* protocol ensures that each capsule signals mostly to a single parent capsule, i.e., the one whose output harmonizes well with its own, which amplifies plausible relationships between capsules and, consequently, between their entities (Sabour et al, 2017).

### 3.4 Software implementation

In terms of software implementation, three main programming languages, namely, Python, R and Matlab are frequently used in omics analysis. Currently, Python is coming to the fore in machine learning in general (Srinath, 2017). Despite many Python innovations, R offers numerous libraries and packages for biological analyses, including ones specifically for handling omics data (Chong and Xia, 2018; Picart-Armada et al., 2018). This is mainly due to the history of bioinformatics analysis using the Bioconductor repository (Gentleman et al., 2005). Nevertheless, we must point out that R has its original roots in statistical analysis. Thus, R also offers methods developed at the borderline between computer science and statistics (Torsten Hothorn, 2022).

The main difference in software implementations using Python or R is usually the target application. Mostly, R packages are created and tested for one data type with very specific properties, see Supplementary Table S1. As a result, the R language in omics analysis is seldomly used directly for developing neural networks, but rather for optimizing more classical learning methods such as linear regression or Bayesian methods. In addition, a large part of scientific research regarding ML algorithms is conducted in Matlab. Nowadays, Matlab also offers many new innovations related mostly to training and proper optimization of error functions in neural networks.

**FIGURE 3:**
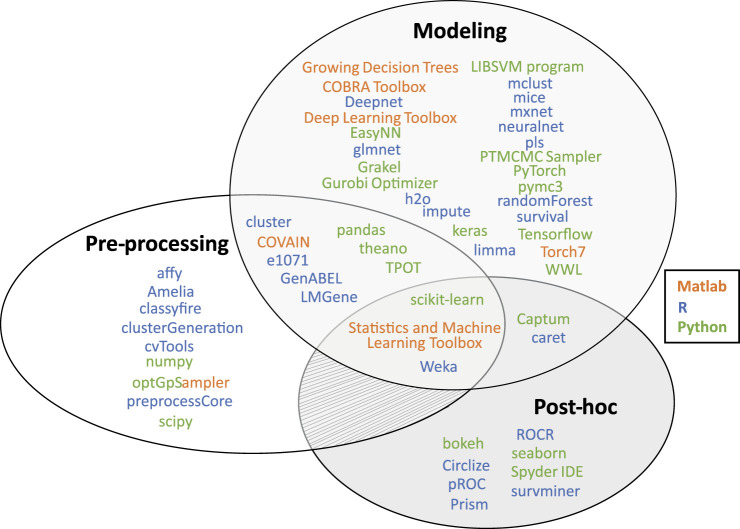
Overview of useful software packages for machine learning implementations from the most prevalent programming languages in computational biology (i.e., R, Python, and Matlab). All listed packages have been applied in an omics data analysis context (see Supplementary Table S1 for references). Most packages focus on either data pre-processing, the modeling phase (i.e., model-based interpretations and designing, training or executing a ML model in general), or the post-hoc analysis phase (i.e., post-hoc interpretations and data visualization).

A combination of different languages also offers more analysis options. Appropriate interfaces exist for example to use Python in R^1^ and Matlab^2^. A summary of useful software packages for (interpretable) machine learning can be found in Figure 3 and Supplementary Table S1.

## 4 What is interpretability?

### 4.1 Basic concepts of interpretability

The concept of interpretability has been thoroughly discussed in recent years in the machine learning community, leading to a diversity of different perceptions, terms, and attempts at its definition (Lipton, 2016; Murdoch et al., 2019; Barredo Arrieta et al., 2020). Terms that are strongly associated with interpretable machine learning are *transparency* (Lipton, 2016; Barredo Arrieta et al., 2020), *white-box* (Loyola-Gonzalez, 2019), *explainability*, *understandability*, and *comprehensibility* (Barredo Arrieta et al., 2020). While all of these terms might capture different notions of the same overall concept (Lipton, 2016; Barredo Arrieta et al., 2020), they seem to refer to the same underlying desires, which are to trust, understand, or interpret the decision-making process or the results obtained from a machine learning model. Besides its controversial nature, there is a strong agreement that the topic of interpretability is important in machine learning (Lipton, 2016; Barredo Arrieta et al., 2020), especially for experts and scientists that deploy ML models to real-world problems (Murdoch et al., 2019).

Due to its many facets, it is necessary to fix a definition of interpretability when writing about it (Lipton, 2016). Interpretability can be defined as “the ability to explain or to provide the meaning in understandable terms to a human” (Barredo Arrieta et al., 2020) or to be able to extract “relevant knowledge from a machine-learning model concerning relationships either contained in data or learned by the model” (Murdoch et al., 2019). In this review, we would like to adapt the second definition and define it in the context of this work as the ability to generate biological insight from data with the help of machine learning methods.

#### 4.1.1 Reliability of interpretations

To gain real insight, any information we extract from a ML model and interpret needs to be reliable. As Murdoch et al. (2019) describe, this depends on two criteria: *predictive accuracy*, i.e., performance of the model, and *descriptive accuracy*, i.e., performance of the interpretation method. They argue that interpretations would be unreliable if either the ML model fails to model the data accurately or the interpretation method is unable to correctly extract information from the model. Furthermore, we argue that interpretability relies on every step that leads towards an interpretation. This includes the whole analysis framework: we need to trust 1) that the raw data contains the desired information in an unbiased manner, 2) that the data preprocessing steps retain the relevant information from the raw data, 3) that, as suggested by Murdoch et al. (2019), the ML model correctly captures relevant information from the training data, and 4) that the interpretation method effectively conveys this information.

All of these points need to work correctly to avoid misleading interpretations. In particular, raw data quality is very important. If the raw data is flawed, both predictions and interpretations will automatically be inaccurate/misleading. Raw data quality relies on the experimental procedure, a topic we hardly touch on in this review. This further demonstrates the broad scope of interpretability.

Preprocessing depends on the properties of the available data, the problem of interest, and the ML model. Thus, individual preprocessing steps might need to be validated for every implementation. Generally, it is crucial to not accidentally lose valuable information during preprocessing, as discussed in Section 2.2.

Regarding the ML model, Murdoch et al. (2019) emphasize that “one must appropriately measure predictive accuracy.” For this, samples in the test set must not be involved in model optimization and training, since they simulate how the model would predict labels of new/unknown samples. Further, one should collect test samples without bias, slight changes in the training set and model should not heavily impact predictive accuracy, and predictions should be equally accurate for all types of samples (Murdoch et al., 2019).

Murdoch et al. (2019) suggest that descriptive accuracy depends on the interpretation and ML method and that some ML methods offer either superior descriptive or predictive accuracy: while, e.g., a deep neural network may outperform a decision tree, the decision tree may be easier to interpret. In systems biology, we frequently want to achieve both, e.g., correctly diagnose a disease and understand the reasoning behind the diagnosis. Therefore, we may have to balance the two objectives (Murdoch et al., 2019).

#### 4.1.2 Interpretation methods

**FIGURE 4:**
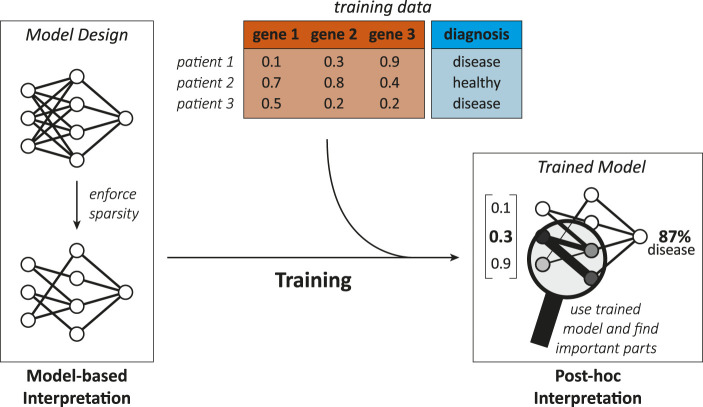
Illustration showing the difference between model-based and post-hoc interpretation methods. A model-based interpretation strategy could be to design a sparse model by limiting the possible connections in a neural network (e.g., with knowledge about biological networks). Once the ML model is trained, post-hoc analysis can reveal the model parts that are most important for predictions, hinting on genes or biological interactions relevant for the disease.

There are two general classes of interpretation methods, namely *post-hoc* (Lipton, 2016; Murdoch et al., 2019; Barredo Arrieta et al., 2020) and *model-based* techniques (Murdoch et al., 2019), which Figure 4 exemplifies. Model-based interpretations rely on the implementation of ML models “readily providing insight into the relationships they have learned” (Murdoch et al., 2019), whereas post-hoc interpretations only take place after the designing and training process and try to produce relevant biological knowledge just from the finalized model (Murdoch et al., 2019; Barredo Arrieta et al., 2020).

##### Model-based interpretation methods

Model-based interpretability can be achieved by enforcing three different properties in a model: “sparsity,” “simulatability,” and “modularity” (Murdoch et al., 2019).

*Sparsity* arises when some parameters are set to zero by the ML model itself or explicitly by the designer with prior knowledge, thereby decreasing the number of variables that need to be comprehended (Murdoch et al., 2019). Further, sparsity can associate parts of the ML model with biological entities, which allows additional interpretations and is discussed in Section 4.4.1. Methods that enforce sparsity require that there is indeed only a limited number of relevant connections between the features and the prediction target as indicated by Murdoch et al. (2019). When too many or the wrong parameters are eliminated, the model might learn an inaccurate/misleading relation. Additionally, any parameter that influences an interpretation should have similar values when we retrain the model with a slightly different training set (Murdoch et al., 2019), e.g., one where a single sample was changed or omitted/added. This requirement is generally known as *stability* in learning theory (Bousquet and Elisseeff, 2002). Methods that offer sparsity are, for instance, lasso regularized models (Murdoch et al., 2019) and nearest shrunken centroid because they intrinsically eliminate contributions of unimportant features.

*Simulatability* refers to the degree at which a person can comprehend and could theoretically think/run through the whole procedure of computing an output for a given input (Murdoch et al., 2019; Barredo Arrieta et al., 2020) “in reasonable time” (Lipton, 2016). Human comprehension demands that the following properties are sufficiently low: the complexity of the studied problem [referred to as the complexity of “the underlying relationship” by Murdoch et al. (2019)], the samples’ dimension (Murdoch et al., 2019), the model’s overall complexity, and the number of steps from input to output (Lipton, 2016). Therefore, making simulatability a requirement would drastically shrink the space of available methods and biological problems (Murdoch et al., 2019). Examples of models that usually exhibit a high level of simulatability are linear and logistic regression models, single decision trees, k-nearest neighbor classifiers, rule-based models, single neuron neural networks (Barredo Arrieta et al., 2020), and linear genetic programs (LGPs).

*Modularity* is a property where the model includes elements (i.e., “modules”) that make the model partially understandable because they are interpretable on their own (Murdoch et al., 2019). In the two case studies of modular designs (Kim and Tagkopoulos, 2018; Alghamdi et al., 2021) we highlight later, modules allow restricted insight because their inputs and outputs are biologically meaningful. Consequently, the module as a whole depicts a biological mechanism. It is the biological process that connects transparent input and output [e.g., transcription and its regulation; translating the genotype and environmental context to the transcriptome (Kim et al., 2016)]. Nonetheless, the way a module mathematically models a biological process could be elusive. This type of modularity seems related to what Lipton (2016) and Barredo Arrieta et al. (2020) call *decomposability*, which they describe as that the model is fully composed of elements (i.e., features, internal variables, computations) that make instinctively sense. Hence, we might call these cases partially decomposable. Neural network based models with a modular design and “generalized additive models” offer modularity (Murdoch et al., 2019), while decision trees and linear models can be fully decomposable (Lipton, 2016).

##### Post-hoc interpretation methods

Post-hoc interpretation techniques act after training and aim to reveal some of the hidden “relationships” the model has internalized by viewing the training samples (Murdoch et al., 2019). We see post-hoc interpretations more generally as the action of extracting valuable information from a trained model. Thus, a post-hoc interpretation could be as simple as communicating naturally meaningful coefficients of a linear model to a human interpreter. There exist various post-hoc approaches for different ML models that try to interpret a trained model by, e.g., assessing the importance of input features or relationships between them (Murdoch et al., 2019), visualizations, providing exemplary predictions, simplifying the model, putting reasonings into words, or elucidating individual properties of the model (Barredo Arrieta et al., 2020).

For additional examples, and further clarifications on the mentioned terms regarding interpretability great resources are the works of Lipton (2016), Murdoch et al. (2019), and Barredo Arrieta et al. (2020).

### 4.2 Interpretability categorization scheme

In this work, we have developed a scheme which allows us to categorize research studies that applied ML models to biological data sets. In this scheme, studies are classified into a total of nine combined categories according to two criteria, 1) the used interpretation method and 2) if and at which point prior biological insight was incorporated into the project. Table 2 summarizes similarities between the reviewed studies in these two characteristics and states the corresponding categorizations.

**TABLE 2:** Categorization of research studies applying machine learning techniques to non-sequential omics data sets. Summary of interpretation methods, assigned category and the approach demonstrated in the publication that led to this classification (*top*). Summary of utilized modeling frameworks, assigned category and prior knowledge that entered the modeling framework (*bottom*).

**Interpretation Method**		
**Approach**	**Category**	**Ref**
Sparse model	model-based	Koh et al. (2019); Pai et al. (2019); Nguyen et al. (2021); Wang et al. (2021)
Modular design	model-based	Kim et al. (2016); Alghamdi et al. (2021)
Well-simulatable model	model-based	Andreozzi et al. (2016); Hu et al. (2018); Sha et al. (2021)
Input-response analysis	post-hoc	Alakwaa et al. (2018); Costello and Martin (2018); Wang et al. (2020); Zhang et al. (2021)
Feature importance	post-hoc	Leitner et al. (2017); Alakwaa et al. (2018); Asakura et al. (2018); Date and Kikuchi (2018); Bahado-Singh et al. (2019); Culley et al. (2020); van Dooijeweert et al. (2021)
	no interpretation methods	Hoehenwarter et al. (2011); Liu et al. (2017); Trainor et al. (2017); Mendez et al. (2019); Sharma et al. (2019); Stamate et al. (2019); Toubiana et al. (2019)

**Modeling Framework**		
**Incorporated Prior Knowledge**	**Category**	**Ref**
Biological network information		
Transcriptional regulatory network	light gray-box	Kim et al. (2016) ^a^; Koh et al. (2019); Nguyen et al. (2021) ^a^; Wang et al. (2021) ^a^
Protein-protein interaction network	light gray-box	Kim et al. (2016); Koh et al. (2019); Wang et al. (2021) ^a^
Co-expression protein network	light gray-box	Kim et al. (2016)
Metabolic network	light gray-box	Alghamdi et al. (2021) ^a^
Pathways of metabolites	dark gray-box	Toubiana et al. (2019) ^b^
Other biological relationships		
Chromosomal allocation of CpG sites	light gray-box	Zhang et al. (2021) ^a^
Expression quantitative trait loci	light gray-box	Nguyen et al. (2021) ^a^
Chemical composition	light gray-box	Alghamdi et al. (2021)
Constraint-based metabolic modeling	light gray-box	Kim et al. (2016) ^b^
	dark gray-box	Andreozzi et al. (2016) ^b^; Culley et al. (2020) ^b^
Reaction kinetics	dark gray-box	Andreozzi et al. (2016) ^b^
No prior knowledge	black-box	Hu et al. (2018); Sha et al. (2021); Alakwaa et al. (2018); Date and Kikuchi (2018); Bahado-Singh et al. (2019), Wang et al. (2020); van Dooijeweert et al. (2021); Leitner et al. (2017); Costello and Martin (2018); Asakura et al. (2018); Sharma et al. (2019); Mendez et al. (2019); Stamate et al. (2019); Liu et al. (2017); Trainor et al. (2017); Hoehenwarter et al. (2011)

a Knowledge was used to select connections in a neural network

b Knowledge was used to create new features/variables.

#### 4.2.1 Use of interpretation methods

Following the definitions laid out in Section 4.1 we differentiate between:

• **No interpretation methods.** Studies that do not implement post-hoc or model-based interpretation methods.
• **Post-hoc interpretations.** Studies that gain biological insight by analyzing a trained ML model with post-hoc interpretation methods.
• **Model-based interpretations.** Studies that gain biological insight by either using a *well-interpretable* machine learning model as their primary model or modifying a machine learning model such that its sparsity, simulatability, modularity or decomposability is increased.

We consider machine learning models to be “well-interpretable” if they were explicitly declared to frequently demonstrate sparsity, simulatability, modularity, or decomposability by the interpretable machine learning community, or if they obviously display one of these properties. In particular, this includes, methods that use lasso regularization or “sparse coding” (Murdoch et al., 2019), decision trees (Lipton, 2016; Murdoch et al., 2019; Barredo Arrieta et al., 2020), linear regression models, logistic regression models, k-nearest neighbor classifiers, single neuron neural networks, rule-based models, Bayesian models (Barredo Arrieta et al., 2020), generalized additive models (Murdoch et al., 2019; Barredo Arrieta et al., 2020), neural network based models with a modular design (Murdoch et al., 2019), nearest shrunken centroid, and linear genetic programs.

#### 4.2.2 Use of prior knowledge

Using prior knowledge to guide the design of a ML model can boost interpretability and even performance, e.g., when introducing sparsity (Murdoch et al., 2019). If neural networks are wired according to known biological relationships, elements of the ML model can be virtually coupled to biological entities. This possibility was demonstrated for cellular components (Ma et al., 2018), genes (Nguyen et al., 2021), regulatory proteins, and protein interaction clusters (Wang et al., 2021). For defining categories with respect to the integration of prior biological knowledge, we adopt a view from the field of system identification (SI). SI discriminates between the three categories black-box, gray-box and white-box for mathematical models based on the amount of theoretical and experimental knowledge that went into their construction (Sjöberg et al., 1995; Isermann and Münchhof, 2011).

Machine learning models are often tightly embedded into a much larger *modeling framework*. This modeling framework includes all data preprocessing steps as explained in Section 2.2 and can be seen as anything that supports the data flow from the initial raw data to a final prediction. Sometimes, this modeling framework can be enormous (Andreozzi et al., 2016), representing a significant portion of the added scientific value of a study. Because prior knowledge can enter not only in the ML model itself but also during preprocessing, we want to utilize this categorization criterion to capture a property of the modeling framework. With this in mind, we differentiate between:

• **Black-box.** Modeling frameworks that do not incorporate any prior biological knowledge—they are purely determined by measurement data (“data-driven”).
• **Dark gray-box.** Modeling frameworks that incorporate prior biological knowledge in any step before the machine learning model that makes the final prediction.
• **Light gray-box.** Modeling frameworks that incorporate prior biological knowledge into their machine learning model. This category also includes cases where prior biological knowledge enters at both points, before the machine learning model, and within it.

Please note that because of how SI (Sjöberg et al., 1995; Isermann and Münchhof, 2011) defines “white-box” models, a corresponding category would inherently exclude any approach that includes a ML model. This is because in SI, the term white-box describes models in which every mechanism and parameter is known from theoretical knowledge (i.e., previous experience and first principles), without relying on any measurement data (Sjöberg et al., 1995). In machine learning, a learning algorithm automatically integrates measurement data into mathematical models, which contradicts with the white-box definition from SI. Consequently, a white-box category does not appear in our scheme. Please further consider that the terms “black-box” and “white-box” frequently pop up in the machine learning literature and try to convey the level of interpretability of a ML model (Lipton, 2016; Loyola-Gonzalez, 2019; Murdoch et al., 2019; Barredo Arrieta et al., 2020). However, we avoid these notions because they seem vaguely defined and overused. We want to emphasize that they should not be confused with the well-established homonyms found in SI (Sjöberg et al., 1995; Ljung et al., 1998; Isermann and Münchhof, 2011) upon which we base our second criterion.

#### 4.2.3 Additional considerations and examples

Although we try to outline clear categories, it is possible to encounter studies whose allocation seems uncertain. In this section, we provide additional considerations together with examples to make assignments more conclusive.

The model-based interpretations category does not exclude the use of post-hoc interpretation methods. From the fact that data is the target of interpretations (Murdoch et al., 2019) and how we defined post-hoc methods follows that post-hoc interpretations must always accompany a model-based strategy. For instance, the post-hoc method *integrated gradients* (Sundararajan et al., 2017) is applied by Nguyen et al. (2021) to a ML model that was modified to exhibit sparsity.

Whether a machine learning model is well-interpretable is difficult to judge. For instance, the notion of simulatability depends on the complexity of the model (Lipton, 2016; Murdoch et al., 2019; Barredo Arrieta et al., 2020). Decomposability demands that all features are meaningful (Lipton, 2016; Barredo Arrieta et al., 2020), which depends on the raw data and preprocessing. For modular designs, individual modules need to be interpretable on their own (Murdoch et al., 2019), which depends on their nature, context, and relationship to each other. Reducing the number of variables that need to be comprehended by building sparse models (Murdoch et al., 2019) might generally improve interpretability. However, if too many variables remain in the sparse model, interpretations may still be limited, as implied by Murdoch et al. (2019). All these factors vary between implementations of the same general ML method. To judge if a ML model is well-interpretable we have taken an Occam’s Razor approach. We assume every implementation of a ML method is well-interpretable if the interpretable machine learning community (Lipton, 2016; Murdoch et al., 2019; Barredo Arrieta et al., 2020) mentions that the method usually displays sparsity, simulatability, modularity, or decomposability.

All categories that assess aspects of the machine learning method are based on *primary ML models*. Many studies develop a machine learning approach and then compare it to a set of well-established base-line models (Asakura et al., 2018; Date and Kikuchi, 2018; Wang et al., 2020; Sha et al., 2021; Zhang et al., 2021). We call the models that the authors present as their methods of choice/interest (or which they primarily use) for predictions the primary ML models. We considered interpretability aspects only of primary models and viewed the modeling framework from their perspective. Consequently, any additional ML models, e.g., base-line models, lasso regression to select features for a primary model (Leitner et al., 2017; Liu et al., 2017; Pai et al., 2019) or kNN for data imputation (Alakwaa et al., 2018; Stamate et al., 2019) did not influence our categorizations.

We considered models whose individual predictions were combined (Kim et al., 2016) as one large primary model. On the other hand, if models receive the same input but predict different target variables (Costello and Martin, 2018) these were not seen as one model.

Biological insight that is a direct consequence of a prediction was not considered to be generated by an interpretation method. For example, Toubiana et al. (2019) mapped pathways onto correlation networks, derived graph-based features and used these to predict if the pathways are part of the tomato metabolism. By repeating this procedure with unlabeled pathways testable hypotheses about their affiliation to tomato can be proposed (Toubiana et al., 2019). Here, the output is directly subject to interpretation, while the model itself is left untouched. We did not consider this case to be an interpretation method. Hypothetically, any prediction made by a ML model could be experimentally tested as long as the output has a biological meaning.

With all of this in mind, we are now ready to highlight some of the works we have categorized in Table 2 in more detail in the next sections. These sections focus on post-hoc and model-based interpretation methods. If studies have integrated prior biological knowledge in an original way, this will also be discussed.

### 4.3 Post-hoc interpretations

#### 4.3.1 Discovering biomarkers by simple feature importance measures

Probably the most frequent approach to extract knowledge from a ML model is to assess *feature importance* in some way. Knowing how individual genes or metabolites influence the predicted probability of a disease can provide a first glimpse into the mechanisms of the disease. Investigating in which biological subsystems (e.g., pathways) predictive genes or metabolites participate lets us narrow down the origin of the disease within the system. A proposed set of relevant molecules could serve as biomarkers, enabling us to develop diagnostic tools that do not require untargeted omics screens. Further, reducing the number of considered variables may lead to more accurate predictions by lowering the noise brought by unnecessary information (Culley et al., 2020). In order to gain biological insight with feature importance scores, the inputs need to have a clear connection to a biological entity. For instance, when working with principal components as inputs, which could be linear combinations of measurements from over 60,000 genes, then, their relative importances most certainly provide no immediate biological insight. Nonetheless, in this specific case, importance scores could be backtraced to meaningful raw features (i.e., genes) by knowing the PCA loadings.

An advantage of methods that evaluate feature importance is that they are convenient to implement, as they come with many software packages for machine learning. Bahado-Singh et al. (2019) used functions from the packages *caret* and *h2o* in R to score patient properties, including metabolomic and proteomic measurements, according to their ability to discriminate between clinical outcomes. This allowed them to propose a single metabolite as a promising biomarker for premature delivery in pregnant women with the same physiology.

Leitner et al. (2017) ranked untargeted metabolomic features according to their importance in a PLS-DA model. The top-ranked metabolite in this analysis pointed them toward a specific metabolic pathway. Experimentally targeting this pathway by Stable Isotope Diluted Direct Infusion Electrospray Ionisation Mass Spectrometry (SID-MS) yielded new metabolomic features that improved predictions with a SVM model when combined with untargeted features. This demonstrates that novel biological insight from interpretations can also allow us to build better predictive models.

**FIGURE 5:**
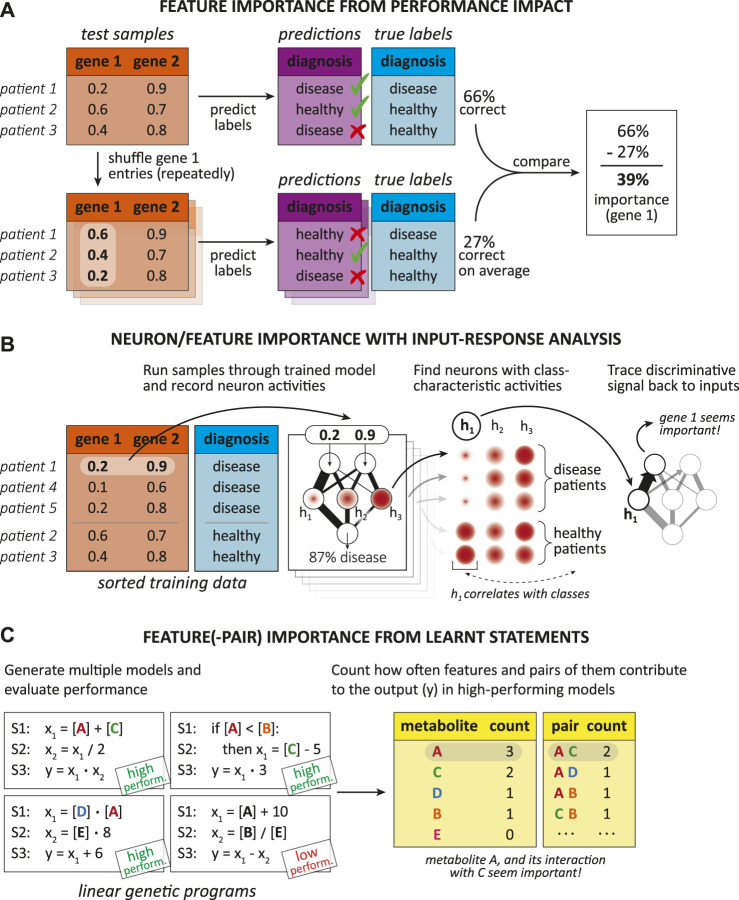
Examples of post-hoc interpretation methods from Section 4.3 in simplified form. **(A)** The impact of perturbing individual features on the overall performance of the ML model can indicate how important features are. This is the basic principle behind “mean decrease accuracy,” as it was performed by Date and Kikuchi (2018). Thereby, entries of individual features are shuffled between test samples multiple times. Model performance is measured each time and compared to the performance obtained by using the unchanged test set. **(B)** Activation patterns of neurons can allow insight into the ML model, revealing important neurons that have learnt to discriminate between classes and important features that enable this discriminative ability. This input-response analysis was part of the post-hoc interpretation strategy of Alakwaa et al. (2018). They used it to verify that some neurons have learnt to distinguish between two cancer subclasses and to discover metabolites that are primarily associated with one subclass. In the generalized version illustrated here, activities of neurons in the first hidden layer are recorded while the already trained neural network processes the training samples. Comparing the activities between different classes can then identify characteristic neurons. Inputs that strongly connect to these class-characteristic neurons are likely important. **(C)** Statements found in well-performing linear genetic programs (LGPs) can reveal important input features and might further indicate important feature interactions. This was demonstrated by Hu et al. (2018) and Sha et al. (2021) and is shown here in a simplified way. As described by Hu et al. (2018) and Sha et al. (2021), LGPs are made up of a sequence of statements that convert some input features, [X], to an output variable, y, and are generated by a process similar to biological evolution. Since LGPs do not need to use all features, individual and pairwise counts of features that influence the output in well-performing programs may indicate the importance of features and their relationships (Sha et al., 2021).

Date and Kikuchi (2018) estimated the relevance of metabolic markers in their deep neural network (DNN) in terms of “Mean Decrease Accuracy (MDA).” MDA measures the impact of perturbing an individual feature on the performance of the ML model (Figure 5A). To compute MDA for a feature, the authors compared the original performance of their ML model to multiple cases in which the entries of the feature were shuffled between data samples. A feature whose entries are mixed between samples loses some of its predictive power because the labels stay fixed, disconnecting many entries from their correct label. Multiple such iterations dampen the stochastic effects of random shuffling. Date and Kikuchi (2018) demonstrated that calculated MDA scores were similar among different ML methods and they allowed them to hypothesize about relevant metabolic markers for a sample’s regional origin.

#### 4.3.2 Biological insight from recording how the model responds to different inputs

Since supervised ML models learn how to map their inputs to different desired outputs, they can react very differently to different samples. Apart from the output itself, there are often internal responses that arise while processing a sample. For instance, neurons in neural networks activate differently, capsules in capsule networks couple to their parents differently, decision trees follow different paths to get to a leaf. Although these responses can be quite distinct, we can expect that they are mostly similar for samples with similar labels (e.g., those belonging to the same class). Monitoring these responses can be a handy tool to extract novel biological knowledge from a ML model. We call this general approach *input-response analysis*.

Alakwaa et al. (2018) addressed feature importance with the same method as Bahado-Singh et al. (2019) and additionally identified relevant metabolites and pathways by tracking how individual neurons in a neural network respond when presented with distinct inputs (Figure 5B). They trained their ML model on metabolite measurements from breast cancer patients belonging to the estrogen receptor positive or negative class, which are associated with distinct survival rates. Depending on the input, neurons found in each layer will activate differently. Alakwaa et al. (2018) noticed significant differences amongst the two cancer classes in the responses of some neurons in the first hidden layer of their trained model. By backtracing these discriminative signals over the strongest neuronal connections to the inputs, they could find relevant metabolites. The authors reported that some of these molecules were indicated to be linked to breast cancer by other studies. Finally, they looked at pathways harbouring relevant metabolites to further investigate their role in cancer metabolism. For this purpose, also data of enzymes showing distinct expression levels between the cancer classes was used. This study demonstrates how learnt connection weights together with neuron response patterns can allow a glimpse into the inner workings of a ML method often thought to be incomprehensible.

Wang et al. (2020) implemented a capsule network (see Section 3.3.3 for explanation) to predict a cell’s type based on its single-cell gene expression pattern. They adopted an interpretation strategy very similar to that of Alakwaa et al. (2018). They analyzed how their capsule network responds to samples from different classes and backtracked the observed signals to the inputs of the network (i.e., transcript levels). This enabled the authors to hypothesize about a set of “core genes” typical for every cell class and allowing it to be discriminated from other cell classes. Their model is divided into two parts, a “feature extractor” and the actual capsule network. The feature extractor consists of several neural networks that each aim to find an informative vector description of the expression levels and supply it to a different primary capsule. A process called “Dynamic routing” connects the primary capsules to higher-level capsules (Sabour et al, 2017). In the implementation of Wang et al. (2020), the higher-level capsules each represent a cell class and their activation levels are used to classify a single-cell mRNA sample. During dynamic routing, so-called *coupling coefficients* are calculated that determine the contribution a primary capsule makes to the activity (Sabour et al, 2017) of a “cell type capsule” (Wang et al., 2020). These coupling coefficients depend on the input and the authors computed their mean values for every cell class. This way, they were able to find the primary capsules that received gene expression information that was most valuable for identifying each cell class. Since every primary capsule receives input from a single neural network, analysis of the weights learned by each network could identify genes characteristic of a cell class. Taken together, the work of Wang et al. (2020) further demonstrates that even very complex model architectures that have many parameters can allow the extraction of novel biological insight.

Nguyen et al. (2021) used the method *integrated gradients* (Sundararajan et al., 2017) to assess the relevance of their features (i.e., SNPs and genes). Integrated gradients presents the trained model with a series of artificial inputs that progressively contain more information from a real sample while looking on how the output changes in response (Sundararajan et al., 2017). With their calculated scores, the authors found genes and SNPs that most influenced probability for schizophrenia. Additionally, they designed their neural network such that links between the input and first hidden layer convey a biological interpretation, representing either SNP-gene or gene-gene interactions. Using a method derived from integrated gradients termed *Conductance* (Dhamdhere et al., 2018) together with their special architecture allowed Nguyen et al. (2021) to evaluate also the importance of the biologically meaningful connections in the neural network. They reported that many of their results are supported by literature, and additional data, respectively. Since the step of assigning neural network links to biological interactions involves altering the ML model, this approach falls under model-based interpretation methods and will be discussed in more detail in Section 4.4.1. Implementations of integrated gradients and conductance are available in the Python package Captum.

The work of Costello and Martin (2018) exemplifies how input-output analysis of a trained ML model can provide biological insight that is experimentally testable. The authors trained multiple models to each predict the current rate of change for another metabolite given metabolite and protein abundances at the same time instant. This design was chosen with the hope of challenging traditional kinetic models in their ability to pursue a metabolic system over time. The models were trained with samples from smoothed metabolite and protein trajectories of measured time series from two biotechnologically interesting pathways. Costello and Martin (2018) demonstrated how their models can generate novel biological knowledge. For that, synthetic data of multiple artificial “strains” was created. Each strain differed in how its protein timeseries was generated (i.e., by changing the parameters of hill function expression models). Using their ML models, they predicted potential product yields for each strain to identify proteins whose over-/underexpression influence yield. This analysis was done with partial least squares (PLS) regression. They also demonstrated that even if their ML models were trained only on two experimental data sets, they could exceed the accuracy of a carefully “handcrafted” kinetic model in predicting metabolite trajectories within a pathway. The ML framework of Costello and Martin (2018) as a whole could be argued to be interpretable because of its modular appearance. Every individual ML algorithm receives the same inputs and predicts another quantity, while both inputs (i.e., protein and metabolite levels) and output (i.e., dynamics of a single metabolite) have a clear biological interpretation. Nonetheless, we see their regression models as separate units and not parts of a modular design since their predictions are not combined and they are trained independently. Further, their ML models are very distinct and rather incomprehensible, comprising completely different methods discovered by the software tool TPOT that automatically generates efficient machine learning solutions for a given task (see Supplementary Table S1 for more information on TPOT).

#### 4.3.3 Biological insight from ML methods that are frequently simulatable

Hu et al. (2018) utilized a supervised method that generates so-called *linear genetic programs (LGPs)* (Figure 5C). The authors used it to separate patients with osteoarthritis from healthy individuals based on their metabolome characteristics. As explained by Hu et al. (2018) and Sha et al. (2021), a linear genetic program is a sequence of “statements” that describes how features (i.e., metabolite abundances) should be combined with themselves or with other variables and under which conditions. At the end, a special variable constitutes the output of the program (i.e., chance for osteoarthritis). LGP classifiers are improved by an algorithm that essentially mimics biological evolution (Hu et al., 2018; Sha et al., 2021). After “training,” Hu et al. (2018) evaluated the number of times a metabolite feature was present in one of their best performing models, and how often two metabolites appeared in the same LGP. With this information and the help of graph analysis they identified potential metabolic markers and showed that they correlate in their incidence in the top LGPs.

In their recent study (Sha et al., 2021), Hu et al. applied the same method to discover metabolites that can differentiate between patients with Alzheimer’s Disease (AD), patients with amnestic mild cognitive impairment, and healthy individuals. Many of the top metabolites found to be predictive of AD were also suggested by two other ML methods (i.e., RF and SVM), however, with some discrepancies.

Alakwaa et al. (2018), Wang et al. (2020), and Hu et al. (2018) found their own method to rank features by their predictive power. When extracting such importance scores from a model, comparing the results to those obtained by established methods can be critical. The result, which features are important, should not depend on the utilized method because feature importance should be fundamentally determined by the causal relationships found in the biological process that created the data. Sha et al. (2021) reported that from 20 relevant metabolites found by their method, 10 overlapped with 20 they had identified using another post-hoc interpretation method on another ML model. Although in total 242 metabolites were considered, this could indicate that some of the top-ranked metabolites from one method might not be good biomarkers. Individual linear genetic programs demonstrate high simulatability because they can be read like a piece of computer code as suggested by Sha et al. (2021). However, as demonstrated by the results of Hu et al. (Hu et al., 2018; Sha et al., 2021), LGPs performing well on the same data can be very diverse, using different features and relationships between them. Hence, we note that one should be careful to not over-interpret a single LGP.

Andreozzi et al. (2016) expanded their previously developed “ORACLE” framework by a machine learning part. According to the authors, ORACLE integrates experimental data, including metabolomics and fluxomics, and theoretical knowledge about enzyme kinetics and creates a collection of kinetic models. The aim of their decision tree algorithm “iSCHRUNK” was then to learn from these kinetic models what makes some of them “feasible” and others not. Kinetic models generated by ORACLE were labeled as either feasible or not feasible. Models were considered feasible if they had a locally stable steady state, and matched theoretical knowledge as well as the available experimental data. The parameter values and feasibility label of each kinetic model embody their training data set. After training, the learned “splitting rules” (see Section 3.1.2 for explanation) can be interpreted as kinetic parameter ranges that partition the parameter space. Drawing from a feasible region of the space allowed the authors to discover new parameter sets corresponding to presumably feasible kinetic models.

Since decision trees are frequently outperformed by more complex methods like random forests (Alakwaa et al., 2018; Sharma et al., 2019), the work of Andreozzi et al. (2016) is an excellent example of how supposably sacrificing predictive accuracy by choosing a simple ML model can drastically increase descriptive accuracy. Using a decision tree allowed them to exploit it as a generator for high-quality kinetic models, which could probably not have been done so easily using more complex ML models like neural networks.

### 4.4 Model-based interpretations

As outlined in Section 4.1.2, model-based interpretation techniques rely on modifying the ML algorithm to increase interpretability or choosing a well-interpretable model (Murdoch et al., 2019). Note that the following examples focus on improving interpretability by design choices rather than selecting archetypally interpretable models. A general pattern that can be recognized is that most studies mentioned here couple parts of their ML model to biological entities with the help of biological network information.

#### 4.4.1 Sparse models allow more in-depth interpretations

In their recent study, Wang et al. (2021) enhanced their capsule network that we described earlier in Section 4.3.2 by incorporating prior insight from biological networks. Their ML model was designed to take a single-cell transcriptomics profile as input and predict the type of the corresponding cell. Expression information from all genes (the inputs) was fed into every primary capsule via its own neural network. In this work (Wang et al., 2021), sparsity was enforced because only genes who are regulated by the same transcription factor (TF) or genes whose proteins interact (i.e., participate in the same interaction subnetwork) provide input to the same primary capsule. This way, primary capsules are primed to represent individual TFs or protein interaction clusters. Gene-TF and gene-cluster relationships were inferred from a transcriptional regulatory network (TRN), and a protein-protein interaction (PPI) network, respectively. After training, they applied a similar post-hoc interpretation strategy as in their previous study (Wang et al., 2020). Again, by calculating mean coupling coefficients for every cell type, the relationships between primary capsules and their parents (the “cell type capsules”) could be unraveled. This time, the mean coupling coefficients could be directly interpreted as relevances of individual TFs and protein clusters for classifying a certain cell type. Their results supported their interpretation approach. They reported that important TFs and PPI clusters were predominantly associated with only a single cell type and many of these affiliations were known from literature. Wang et al.’s work demonstrates that with the help of prior knowledge more in-depth interpretations are possible [compare Wang et al. (2020) with Wang et al. (2021)]. Their previous black-box modeling framework was converted to a gray-box and by invoking sparsity they successfully implemented a model-based interpretation method.

**FIGURE 6:**
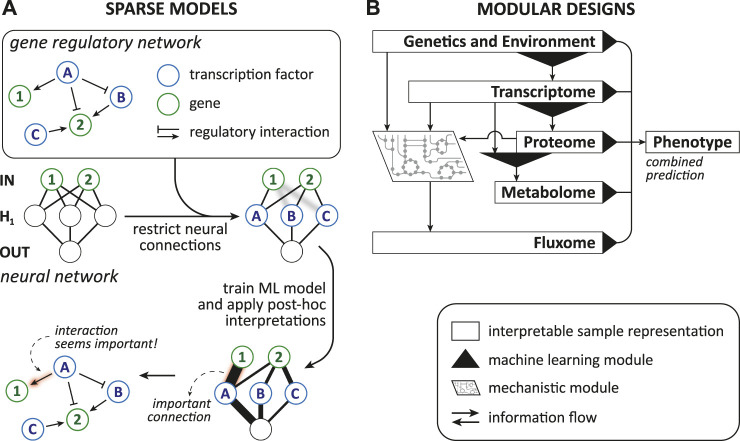
Examples of model-based interpretation methods from Section 4.4 in simplified form. **(A)** Guiding the topology of a neural network by a biological network can increase interpretability. This example captures the fundamental principle of the model-based interpretation strategy presented by Nguyen et al. (2021) and further by Wang et al. (2021). By assigning each neuron in the first hidden layer, H_1_, to a biological entity (i.e., a transcription factor in this example), connections to biologically meaningful inputs (i.e., genes) can be wired according to a biological network. This limits the possible connections in the neural network, introducing sparsity. After training, post-hoc techniques could measure the importance (*red glow*) of the interpretable connections, revealing potentially relevant biological interactions. **(B)** Simplified version of the modular design described by Kim et al. (2016) for predicting the growth phenotype, metabolic dynamics, and expression levels of a cell from its genetics and environment. In general, a modular design may describe a sample or aspects of it in different biologically meaningful ways (i.e., interpretable sample representations). Modules then convert between these transparent representations and may rely on machine learning or mechanistic principles. In the portrayed example (Kim et al., 2016), ML modules connect the genetic and environmental inputs and different omics representations. A mechanistic module (i.e., a metabolic model) is embedded into the design and infers the fluxome under constraints derived from multiple representations. Finally, predictions from multiple ML modules are combined to estimate the phenotype.

Nguyen et al. (2021) deployed a sparse deep neural network to learn about potential biological relationships. Their model infers a diagnosis for schizophrenia from transcriptomics and genetic variants (SNPs) data. Features from both biological data types served as the inputs for the neural network. However, neurons in the first hidden layer were allowed to receive only information from inputs that are associated with the same gene (Figure 6A). This way, these neurons were tied to individual genes similar to the primary capsules in the work of Wang et al. (2021). Associations between the gene neurons and inputs were inferred from expression quantitative trait loci (the gene’s expression is influenced by the input SNP), and transcriptional regulatory interactions (the gene is regulated by the input gene). Since, in their design, the inputs, the first hidden neurons, and connections between them have a biological meaning, more advanced post-hoc interpretations were possible, as described in Section 4.3.2. Additionally, the authors’ neural network was lasso regularized (see Section 3.2.2 for explanation) such that inputs from genes and SNPs with low predictive power are ignored. Both limited connectivity and lasso regularization increase the sparsity of the ML model, making interpretations easier.

Koh et al. (2019) employed a network-focused strategy to classify breast cancer tumors based on their multi-omics signatures and learn about molecular subsystems that characterize tumor subclasses. Raw transcriptomics, proteomics, and gene copy number features were converted to one feature per molecular interaction. Considered interactions were either TF-gene or protein-protein interactions from a TRN, or PPI network, respectively. The new interaction-level features should reflect the probabilities of each interaction and were, thus, calculated such that they were high if both interaction partners were overexpressed, and low if both were underexpressed. Gene copy number information served as a tool to scale mRNA abundances, reducing/increasing them when the corresponding gene was over-/underrepresented. For learning from the new features, the authors modified the original nearest shrunken centroid (NSC) algorithm. As many other supervised methods, vanilla NSC cannot integrate any prior knowledge about how features might influence each other. However, their modification allowed NSC to consider also the features of interactions that are close in the biological network context when deciding whether an interaction’s feature is important for discriminating between classes. This allowed the authors to favor interactions that form subsystems. The authors suggested that these subsystems are biologically more meaningful than important interactions that are dispersed over the biological network. Importantly, NSC chooses a set of relevant features for every class separately (Tibshirani et al., 2002). Consequently, the subsystems discovered by Koh et al. (2019) varied between tumor subclasses. Further, identifying annotated pathways that agree with important subsystems facilitated interpretability and enabled the authors to hypothesize about pathway over-/underexpression in breast cancer subclasses.

The work of Koh et al. (2019) demonstrates that biological expertise can help us to carefully engineer new interpretable features that allow us to view our data from a different (e.g., network) perspective. Notably, the authors reported that in comparison to unmodified NSC applied directly on proteomics and transcriptomics features, their method performed worse on experimental data and better on synthetic data. Although their interaction-level features seem to have captured most of the valuable information stored in the raw features while offering great interpretability, this example again emphasizes that one should be careful when replacing original features, as mentioned earlier in Section 2.2.

#### 4.4.2 Modular designs are partially transparent

Kim et al. (2016) curated a large data compendium for *E. coli* “Ecomics,” which harbours measurements from five different omics layers, together with data about the experiments and network-type data. With this data collection they predicted the complete state (i.e., levels of mRNA, proteins, metabolites, and metabolic fluxes) and growth dynamics of a cell based on its genetics (i.e., strain, genetic perturbations) and environmental factors (i.e., medium, stress). Their design (Figure 6B) was divided into modules that each predict quantities from only one omics layer. The metabolic fluxes were predicted with constraint-based metabolic modeling, while all other modules used machine learning (i.e., a recurrent neural network or lasso regression). Modules were partly exchanging information, providing and/or receiving predicted values to/from other modules. Information from all modules was compiled to collectively predict growth rate. Most interesting for this review is their recurrent neural network (RNN) for predicting transcript abundances. The RNN received a description of the experimental condition and was trained to match experimental transcript profiles with its predictions. The authors selected a RNN for this task to account for cycles (i.e., “feedback loops”) frequently found in transcriptional regulatory networks. The authors hoped that signals would propagate through the iterative layers of the RNN similar to signals traveling in a loop in the biological network. Further, they chose a sigmoid activation function partly because of its similarity to the Hill function. Intriguingly, when neural connections (referred to as “network topology” by the authors) in the RNN were guided by a transcriptional regulatory network, predictions were more accurate. As a whole their modeling framework is a good example of an interpretable machine learning framework due to its pronounced modularity. Every input and output of a module has a clear biological meaning. Besides providing transparency, modular designs have the advantage that modules can be trained/tuned independently as long as data for a module’s input and output is available, which allowed Kim et al. (2016) to use most of their data collection as training data. Further, they integrated prior knowledge at several points in their design, including the metabolic model for fluxome predictions. This makes their modeling framework a light gray-box.

Alghamdi et al. (2021) developed a graph neural network that can estimate metabolic fluxes in one cell from single-cell transcriptomics data. For that the metabolic network was viewed as a directed factor graph (i.e., a special bigraph). In this bigraph, “factor nodes” were individual metabolites and “variable nodes” embodied the reactions in which connected metabolites participate. Directed links indicated whether the metabolite acts as a product or a substrate in a reaction. This graph was constructed from the stoichiometry of a global metabolic network, and then reduced in size to cope with the computational cost linked to finding global flux solutions. Reductions were realized by combining reactions and omitting certain metabolites. To train their model a tailored loss function (see Section 3 for explanation) was designed. Therein reasonable solutions were defined to minimize the “flux imbalance” (i.e., influx versus outflux) of all metabolites, harbour zero or positive fluxes with an appropriate scale, and possess consistency with experimental data. Each rate of a combined reaction was estimated from the transciptomic features of its associated genes via a deep neural network (DNN), resulting in a total of 169 parallel DNNs that need to be trained in harmony. For training, Alghamdi et al. (2021) used their own algorithm. They tested their approach on various data sets, including their own, where they compared predicted flux changes due to genetic and environmental perturbations with experimentally observed metabolite concentration changes, confirming the predictive ability of their approach. We see their complete graph neural network as a modular system with good model-based interpretability. Every input (i.e., single-cell transcriptomic features) and output (i.e., metabolic fluxes) of each DNN module has a clear biological interpretation. Modules are arranged/connected according to a biological network topology, allowing network analysis. This possibility was demonstrated by the authors: by specifically up- or downregulating groups of genes (e.g., in glycolysis) certain metabolic subnetworks (e.g., the Krebs cycle) were impacted as expected. Further, they showed that targeting individual genes can reveal the genes that most influence certain fluxes.

## 5 Conclusions and outlook

In this review, we have categorized 26 scientific papers according to their interpretation strategies and the integration of prior knowledge and discussed some of them in detail. We have found that despite the large diversity of machine learning methods utilized in these studies, some parallels in their interpretation methods can be established. The majority of studies computed scores that assess the importance of input features (Alakwaa et al., 2018; Asakura et al., 2018; Date and Kikuchi, 2018; Hu et al., 2018; Bahado-Singh et al., 2019; Koh et al., 2019; Wang et al., 2020; Nguyen et al., 2021; Sha et al., 2021; Wang et al., 2021). These scores were then sometimes used to discover molecular subsystems (e.g., pathways) of interest (Alakwaa et al., 2018; Koh et al., 2019; Wang et al., 2020; Nguyen et al., 2021). Most model-based interpretation methods relied on either coupling parts of a machine learning model to comprehensible biological entities [e.g., genes (Nguyen et al., 2021), TFs (Wang et al., 2021), interacting proteins (Wang et al., 2021), fluxes (Alghamdi et al., 2021)] or associations between them [e.g., regulatory interactions (Wang et al., 2021), SNP-gene links (Nguyen et al., 2021)] or implementing ML methods that can be considered simulatable (Andreozzi et al., 2016; Hu et al., 2018; Sha et al., 2021). Many papers integrated prior knowledge in the form of biological networks into their modeling frameworks (Koh et al., 2019; Toubiana et al., 2019; Alghamdi et al., 2021; Nguyen et al., 2021; Wang et al., 2021), thereby turning them into gray-boxes; while some studies even incorporated whole constraint-based models (Kim et al., 2016; Culley et al., 2020). Whenever extracting knowledge from machine learning approaches, it is important to make sure that the results are in-line with available literature. One reason why this is especially critical is that many ML models use stochastic training algorithms that can produce drastically different parameterizations on the same training set. When these parameters then influence interpretation results, e.g., by calculating importance scores, we need to make sure that the results are not due to random effects. In other words, results found by interpretation methods should be consistent between different training runs and methods, to not fall into the trap of overinterpretation.

Because we find that the combinatorial space of distinct biological data sets (in source/type, dimension, and size) and what we could learn from them seems endless, interpretation methods might always need to be tailored to a specific scientific problem. Just like in data preprocessing (see Section 2.2) there is no universal recipe for good results. This is, despite some fundamental similarities, reflected in the diversity of approaches we highlighted in this review. A consequence of this diversity is that putting interpretation strategies into well-defined categories can be complicated. One reason for this is the fuzziness of the notions associated with interpretability. For instance, the definition of simulatability is very subjective. At which point is a ML model like a decision tree simple enough for a human to reconstruct its decision-making process? Apart from the ambiguity in terminology arising from different notions, we see a high relevance of interpretable machine learning in systems biology research.

## Footnotes

1
https://www.rstudio.com/blog/reticulate-r-interface-to-python/

2
http://mathworks.com/help/matlab/call-python-libraries.html
