# Supplementary material for: Interpretable machine learning methods for predictions in systems biology from omics data
Source: Front Mol Biosci. 2022 Oct 17;9:926623. doi: 10.3389/fmolb.2022.926623 (PMC9650551; doi:10.3389/fmolb.2022.926623)
Supplement: Supplementary file 1 [file Table1.pdf]

Supplementary Table S1: Overview of software packages

Interpretable machine learning methods for predictions in systems biology from omics data

David Sidak[1], Jana Schwarzerová[1,2], Wolfram Weckwerth[1,3], and Steffen Waldherr[1,*]

[1]University of Vienna, Molecular Systems Biology (MOSYS), Department of Functional and Evolutionary Ecology, Faculty of Life Sciences, 1030 Vienna, Austria

[2]Department of Biomedical Engineering, Faculty of Electrical Engineering and Communication, Brno University of Technology, Brno, Czech Republic

[3]Vienna Metabolomics Center (VIME), University of Vienna, Faculty of Life Sciences, 1030 Vienna, Austria

[*]Corresponding author: steffen.waldherr@univie.ac.at

Table 1: Brief overview of packages and libraries implementation.

Name package/Toolbox	Programming language	Methods	Phase	Brief description	Use of OMICs data
affy	R	Normalization	Preprocessing	Methods for Affymetrix Oligonucleotide Arrays	DOI: 10.1038/ncomms13090
Amelia	R	Bootstrapping-based algorithm	Preprocessing	A Program for Missing Data	DOI: 10.1016/j.cell.2019.04.016
bokeh	Python	Visualization	Post-hoc	A library for creating interactive visualizations for modern web browsers	DOI: 10.1007/s11306-019-1612-4

Continued on next page

Table 1: continued from previous page

Name package	Programming language	Methods	Phase	Brief description	Use of OMICs data
Captum	Python	Integrated Gradients, Gradient SHAP, DeepLIFT, Guided Back-propagation and Deconvolution, Feature Ablation, etc.,	Modeling / Post-hoc	An extensible library for model interpretability built on PyTorch	DOI: 10.1093/bioinformatics/btaa866
Growing Decision Trees	Matlab	Classification and Regression Tree (CART)	Modeling	Function based on algorithm created decision trees	DOI: 10.1073/pnas.2002959117
caret	R	classification, regression, bagging, multivariate adaptive regression splines, etc.,	Modeling / Post-hoc	Classification and Regression Training; functions for training and plotting classification and regression models. Evaluate feature importance.	Conference abstract: Schwarzerova, J., et al. OMICs prediction for Hordeum vulgare using Random Forest methodology. The Biomania Student Scientific Meeting 2022, Book of abstract. 1st. Brno: Masaryk University Press, 2022. 5252. ISBN: 978-80-280-0040-0.
Circlize	R	Visualization	Post-hoc	Circular layout is an efficient way for the visualization of large amounts of information.	DOI: 10.1021/acs.jproteome. 7b00595
classyfire	R	R Interface to the 'ClassyFire' REST API	Preprocessing	Retrieve existing entity classifications and submit new entities for classification; application for automated structural classification of chemical entities.	DOI: 10.1021/acs.analchem. 7b03795
cluster	R	agglomerative hierarchical clustering, gaverage	Preprocessing / Modeling	Methods for Cluster analysis. It extends the original from Peter Rousseeuw, Anja Struyf and Mia Hubert, based on Kaufman and Rousseeuw (1990) "Finding Groups in Data"	DOI: 10.1016/j.ymeth.2019.03.004

Continued on next page

Table 1: continued from previous page

Name package	Programming language	Methods	Phase	Brief description	Use of OMICs data
clusterGeneration	R	random clusters, covariance/correlation matrices, pairs of clusters or cluster distributions	Preprocessing / Modeling	Random Cluster Generation (with Specified Degree of Separation)	DOI: 10.3390/metabo7020030
COBRA Toolbox	Matlab	Dynamic FBA, MTA, Kullback-Leibler Distance, etc.,	Modeling	Quantitative predictions of cellular and multicellular biochemical networks with constraint-based modelling	DOI: 10.1002/bit.27714
COVAIN	Matlab	Statistics methods such as PCA and ICA, Correlation analysis, differential Jacobian matrix	Preprocessing	Multiomics tool for uni- and multivariate statistics, time-series and correlation network analysis and inverse estimation of the differential Jacobian from metabolomics covariance data	DOI: 10.1007/s11306-012-0399-3
cvTools	R	cross-validation	Preprocessing	Functions for cross-validation with minimal programming effort and assist users with model selection.	DOI: 10.3390/metabo7020030
Deepnet	R	Deep learning,	Modeling	Deep learning toolkit including implementations of deep learning architectures and neural network algorithms.	Conference abstract: LIANG, Christine A., et al. Proteomics analysis of FLT3ITD mutation in acute myeloid leukemia using deep learning neural network. Annals of Clinical & Laboratory Science, 2019, 49.1: 119-126.
Deep Learning Toolbox	Matlab	Deep learning	Modeling	It provides a framework for designing and implementing deep neural networks with algorithms, pretrained models, and apps.	DOI: 10.1038/s41598-019-47765-6

Continued on next page

Table 1: continued from previous page

Name package	Programming language	Methods	Phase	Brief description	Use of OMICs data
EasyNN	Python	Neural Networks	Modeling	A package designed to provide an easy-to-use Neural Network. It is designed to work out of the box with multiple data sets while allowing the user to customize features.	Conference abstract: LIANG, Christine A., et al. Proteomics analysis of FLT3ITD mutation in acute myeloid leukemia using deep learning neural network. Annals of Clinical & Laboratory Science, 2019, 49.1: 119-126.
e1071	R	short time Fourier transform, fuzzy clustering, support vector machines, shortest path computation, bagged clustering, naive Bayes classifier, generalized k-nearest neighbour	Preprocessing / Modeling	Library focusing on misc functions of statistics and probability theory group (Formerly: E1071)	DOI: 10.1021/acs.analchem7b03795 10.1016/j.aca.2018.02.045
GenABEL	R	computational genetics, GWAS	Preprocessing / Modeling	A package for genome-wide association analysis between quantitative or binary traits and single-nucleotide polymorphisms	DOI: 10.1007/s11306-017-1239-2

Continued on next page

Table 1: continued from previous page

Name package	Programming language	Methods	Phase	Brief description	Use of OMICs data
glmnet	R	LASSO, linear regression, logistic and multinomial regression modeling, Poisson regression, Cox modeling, multiple-response Gaussian, and the grouped multinomial regression	Modeling	Extremely efficient procedures for fitting the entire lasso or elastic-net regularization path for linear regression, logistic and multinomial regression models, Poisson regression, Cox model, multiple-response Gaussian, and the grouped multinomial regression.	DOI: 10.1007/s11306-017-1239-2
Grakel	Python	Graph kernels	Modeling	An implementations of several well-established graph kernels.	DOI: 10.1093/bioinformatics/btaa655
Gurobi Optimizer	Python	mixed-integer programming, mathematical optimization modeling	Modeling	The Gurobi Python API is mathematical optimization coded modeling	DOI: 10.1016/j.cell.2019.04.016
h2o	R	Generalized Linear Models, Gradient Boosting Machines, Random Forests, Deep Learning, SNaive Bayes, Generalized Additive Models, etc.,	Modeling	Open source machine learning platform that offers parallelized implementations of many supervised and unsupervised machine learning algorithms	DOI: 10.1021/acs.jproteome. 7b00595

Continued on next page

Table 1: continued from previous page

Name package	Programming language	Methods	Phase	Brief description	Use of OMICs data
impute	R	built-in imputation method listed under imputations or create one yourself	Preprocesing	Function performing the imputation on a data set and returns, alongside with the imputed data set, an object which can contain learned coefficients and helpful data. It can then be passed together with a new data set to reimpute.	DOI: 10.1038/ncomms13090
keras	Python	Deep learning	Modeling	Keras offers deep-dives into specific topics such as layer subclassing, fine-tuning, or model saving	DOI: 10.1093/bioinformatics/btab285 10.1038/s42256-020-00244-4 10.1073/pnas.2002959117 10.1016/j.ymeth.2019.03.004 10.1007/s11306-019-1612-4
limma	R	Empirical Bayes model	Modeling	Linear models and differential expression for microarray data	DOI: 10.1016/j.ccell.2020.09.014 10.1038/ncomms13090 10.1021/jproteome.7b00595
LMGene	R	Linear model and glog transformation	Preprocessing / Modeling	LMGene Software for Data Transformation and Identification of Differentially Expressed Genes in Gene Expression Arrays	DOI: 10.1016/j.cell.2019.04.016
LIBSVM program	Python	Support Vector Machines	Modeling	An integrated software for support vector classification, regression and distribution estimation.	DOI: 10.1016/j.cels.2016.03.001

Continued on next page

Table 1: continued from previous page

Name package	Programming language	Methods	Phase	Brief description	Use of OMICs data
mclust	R	Gaussian Mixture Modelling	Modeling	Gaussian finite mixture models fitted via EM algorithm for model-based clustering, classification, and density estimation, including Bayesian regularization, dimension reduction for visualisation, and resampling-based inference.	DOI: 10.1038/ncomms13090
mice	R	logistic regression, polytomous logistic regression, proportional odds	Modeling	Multivariate Imputation by Chained Equations is mice algorithm in which built-in imputation models are provided for continuous data, binary data, unordered categorical data and ordered categorical data.	DOI: 10.1007/s11306-017-1239-2
mxnet	R	Deep learning	Modeling	MXNet offers a straightforward yet powerful interface to utilize deep learning in the R programming language.	DOI: 10.1016/j.aca.2018.02.045
neuralnet	R	Backpropagation, resilient backpropagation with or without weight backtracking	Modeling	Training of neural networks using backpropagation. The package allows flexible settings through custom-choice of error and activation function. Furthermore, the calculation of generalized weights is implemented.	DOI: 10.3390/metabo7020030

Continued on next page

Table 1: continued from previous page

Name package	Programming language	Methods	Phase	Brief description	Use of OMICs data
numpy	Python	Auxiliary functions for scientific computing	Preprocessing	NumPy is the fundamental package for scientific computing in Python.	DOI: 10.1038/s41467-020-18008-4 10.1007/s11306-019-1612-4 10.1038/s41467-020-17910-1
optGpSampler	Matlab / Python	Artificial Centering Hit-and-Run algorithm	Preprocessing	OptGpSampler requires a linear programming solver to find the initial point in the solution space.	DOI: 10.1016/j.cell.2019.04.016
pandas	Python	Auxiliary functions for computing data analysis	Preprocessing / Post-hoc	A fast, powerful, flexible and easy to use open source data analysis and manipulation tool	DOI: 10.1038/s41467-020-18008-4 10.1007/s11306-019-1612-4
pls	R	Multivariate regression methods, Partial Least Squares Regression, Principal Component Regression, Canonical Powered Partial Least Squares	Modeling	Partial Least Squares and Principal Component Regression	DOI: 10.1021/acs.analchem. 7b03795
pROC	R	comparing receiver operating characteristic, area under the curve, statistical tests based on U-statistics or bootstrap	Post-hoc	Display and Analyze ROC Curves	DOI: 10.1021/acs.jproteome. 7b00595
preprocess-Core	R	Quantile Normalization, Background Correction	Preprocessing	A library of core preprocessing routines	DOI: 10.1038/ncomms13090

Continued on next page

Table 1: continued from previous page

Name package	Programming language	Methods	Phase	Brief description	Use of OMICs data
Prism	R	Visualization	Post-hoc	Access Data from the Oregon State Prism Climate Project	DOI: 10.1016/j.cell.2019.04.016
PTMCMC Sampler	Python	Parallel Tempering MCMC code	Modeling	PTMCMCSampler performs MCMC sampling using advanced techniques. The code implements a variety of proposal schemes, including adaptive Metropolis and differential evolution.	DOI: 10.1038/s41467-020-17910-1
PyTorch	Python	Machine learning framework, Deep learning	Modeling	An open source machine learning framework that accelerates the path from research prototyping to production deployment. PyTorch is an optimized tensor library for deep learning using GPUs and CPUs	DOI: 10.1016/j.ccell.2020.09.014 10.1093/bioinformatics/btaa866 10.1109/BIBM.2018.8621345 10.3390/cancers13123047
pymc3	Python	MCMC algorithms, Bayesian nonparametric models	Modeling	Probabilistic Programming in Python	DOI: 10.1038/s41467-020-18008-4
random-Forest	R	Breiman's random forest algorithm	Modeling	Implements random forest algorithm for classification and regression.	DOI: 10.1021/acs.analchem.7b03795
ROCR	R	sensitivity/ specificity curves, recision/recall plots, cross-validation and bootstrapping	Post-hoc	ROCR is easy to use, with only three commands and reasonable default values for all optional parameters.	10.1021/acs.analchem.7b03795

Continued on next page

Table 1: continued from previous page

Name package	Programming language	Methods	Phase	Brief description	Use of OMICs data
scikit-learn	Python	Classification, regression, clustering, dimensionality reduction, model selection	Preprocessing / Modeling / Post-hoc	Simple and efficient tools for predictive data analysis. Built on NumPy, SciPy, and matplotlib.	DOI: 10.1016/j.cell.2019.04.016 10.1186/s12859-021-04209-1 10.1038/s41467-020-18008-4 10.1371/journal.pcbi.1005986
scipy	Python	Optimization, integration, interpolation, eigenvalue problems, algebraic equations, differential equations and statistics	Preprocessing	Fundamental algorithms for scientific computing in Python	DOI: 10.1038/s41467-020-18008-4
seaborn	Python	Visualization	Post-hoc	Statistical data visualization	DOI: 10.1038/s41467-020-18008-4
Spyder IDE	Python	Auxiliary tool	Post-hoc	Spyder is a free and open source scientific environment	DOI: 10.1016/j.cell.2019.04.016
Statistics and Machine Learning Toolbox	Matlab	Classification and Regression Learner apps, or programmatically, using AutoML	Preprocessing / Modeling / Post-hoc	It provides functions and apps to describe, analyze, and model data. You can use descriptive statistics, visualizations, and clustering for exploratory data analysis	DOI: 10.3389/fmolb.2017.00084
survival	R	Multi-state curves, Cox models, and parametric accelerated failure time models	Modeling	Contains the core survival analysis routines.	DOI: 10.1021/acssynbio.0c00129
survminer	R	Visualization	Post-hoc	Contains the function for specification visualization of outputs from survival packages.	DOI: 10.15252/msb.20188497

Continued on next page

Table 1: continued from previous page

Name package	Programming language	Methods	Phase	Brief description	Use of OMICs data
Tensorflow	Python	Machine learning modeling	Modeling	TensorFlow makes it easy for beginners and experts to create machine learning models for desktop, mobile, web, and cloud	DOI: 10.15252/msb.20188497
theano	Python	Optimization method	Preprocessing / Modeling	A library that allows you to define, optimize, and efficiently evaluate mathematical expressions involving multi-dimensional arrays	DOI: 10.1007/s11306-019-1612-4
Torch7	Matlab	Auxiliary tool for machine learning in Matlab	Modeling	A Matlab-like Environment for Machine Learning	DOI: 10.1038/nmeth.4627
TPOT	Python	Optimizing pipeline	Preprocessing / Modeling	TPOT stands for Tree-based Pipeline Optimization Tool. Automates model selection.	DOI: 10.1038/s41540-018-0054-3
Weka	R / Java	pre-processing, classification, regression, clustering, association rules, and visualization	Preprocessing / Modeling / Post-hoc	A collection of machine learning algorithms	DOI: 10.1038/s42003-019-0440-4
WWL	Python	Graph Kernels	Modeling	This repository contains the accompanying code for the NeurIPS 2019 paper Wasserstein Weisfeiler-Lehman Graph Kernels	DOI: 10.1093/bioinformatics/btaa655
