# Supplementary material for: Interpretable machine learning methods for predictions in systems biology from omics data
Source: Front Mol Biosci. 2022 Oct 17;9:926623. doi: 10.3389/fmolb.2022.926623 (PMC9650551; doi:10.3389/fmolb.2022.926623)
Supplement: Supplementary file 2 [file Image1.pdf]

Supplementary Figure S1: Comparison of effective raw data set dimensions and sizes in the categorized studies with references.

Interpretable machine learning methods for predictions in systems biology from omics data

David Sidak[1], Jana Schwarzerová[1,2], Wolfram Weckwerth[1,3], and Steffen Waldherr[1,*]

[1]University of Vienna, Molecular Systems Biology (MOSYS), Department of Functional and Evolutionary Ecology, Faculty of Life Sciences, 1030 Vienna, Austria

[2]Department of Biomedical Engineering, Faculty of Electrical Engineering and Communication, Brno University of Technology, Brno, Czech Republic

[3]Vienna Metabolomics Center (VIME), University of Vienna, Faculty of Life Sciences, 1030 Vienna, Austria

[*]Corresponding author: steffen.waldherr@univie.ac.at

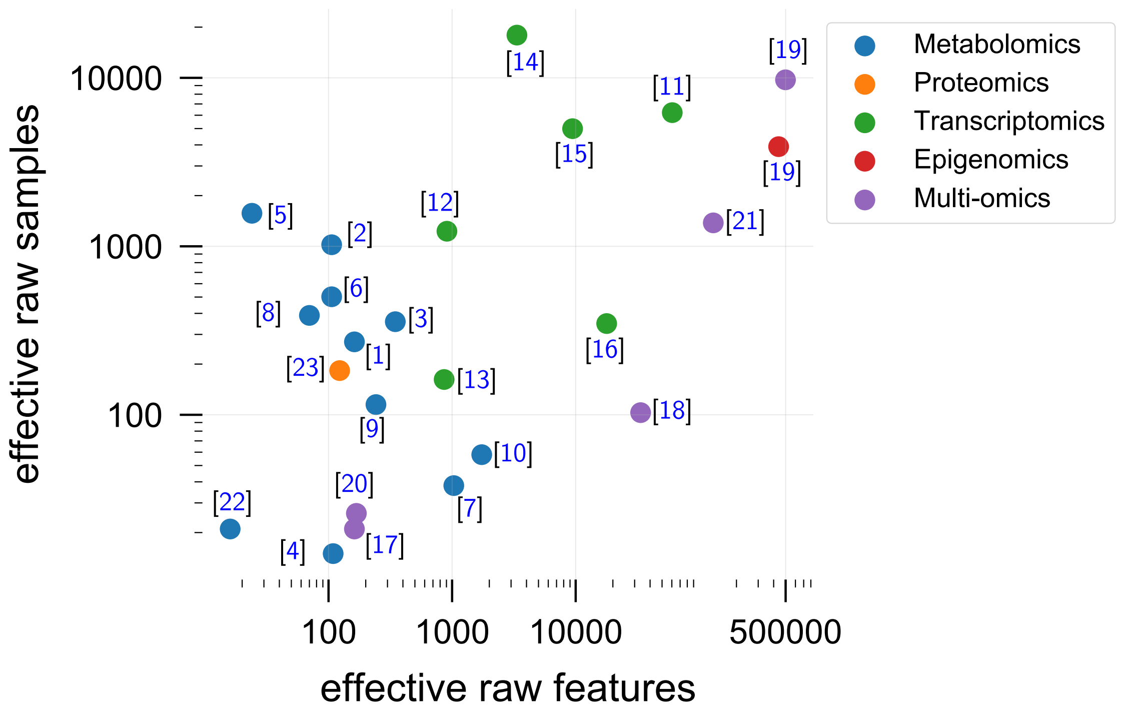

Figure S1: Comparison of effective raw data set dimensions and sizes in the categorized studies with references. Each point represents a data set that was used for optimizing and testing at least one predictive model. In *multi-omics*, a data set includes measurements from multiple omics sources. Each data set is plotted at the position that corresponds to its effective raw dimension and size. Please refer to the main text for explanations on the meaning of effective raw feature and sample counts (Main text, Section 2.1). Note that the graph shows only a selection of all ML-ready data sets from all studies.

References

[1] Alakwaa FM, Chaudhary K, Garmire LX. Deep Learning Accurately Predicts Estrogen Receptor Status in Breast Cancer Metabolomics Data. *Journal of Proteome Research* **17** (2018) 337–347. doi:10.1021/ACS.JPROTEOME.7B00595.

[2] Date Y, Kikuchi J. Application of a Deep Neural Network to Metabolomics Studies and Its Performance in Determining Important Variables. *Analytical Chemistry* **90** (2018) 1805–1810. doi:10.1021/ACS.ANALCHEM.7B03795.

[3] Stamate D, Kim M, Proitsi P, Westwood S, Baird A, Nevado-Holgado A, et al. A metabolite-based machine learning approach to diagnose Alzheimer-type dementia in blood: Results from the European Medical Information Framework for Alzheimer disease biomarker discovery cohort. *Alzheimer's & dementia* (2019). doi:10.1016/j.trci.2019.11.001.

[4] Toubiana D, Puzis R, Wen L, Sikron N, Kurmanbayeva A, Soltabayeva A, et al. Combined network analysis and machine learning allows the prediction of metabolic pathways from tomato metabolomics data. *Communications Biology* **2** (2019) 214. doi:10.1038/s42003-019-0440-4.

[5] Liu J, Semiz S, Van Der Lee SJ, Van Der Spek A, Verhoeven A, Van Klinken JB, et al. Metabolomics based markers predict type 2 diabetes in a 14-year follow-up study. *Metabolomics* **1** (2017) 104. doi:10.1007/s11306-017-1239-2.

[6] Asakura T, Date Y, Kikuchi J. Application of ensemble deep neural network to metabolomics studies. *Analytica Chimica Acta* **1037** (2018) 230–236. doi:10.1016/J.ACA.2018.02.045.

[7] Trainor PJ, de Filippis AP, Rai SN. Evaluation of Classifier Performance for Multiclass Phenotype Discrimination in Untargeted Metabolomics. *Metabolites* **7** (2017). doi:10.3390/METABO7020030.

[8] Hu T, Oksanen K, Zhang W, Randell E, Furey A, Sun G, et al. An evolutionary learning and network approach to identifying key metabolites for osteoarthritis. *PLOS Computational Biology* **14** (2018) e1005986. doi:10.1371/JOURNAL.PCBI.1005986.

[9] Sha C, Cuperlovic-Culf M, Hu T. SMILE: systems metabolomics using interpretable learning and evolution. *BMC Bioinformatics* **22** (2021) 1–17. doi:10.1186/S12859-021-04209-1.

[10] van Dooijeweert B, Broeks MH, van Beers EJ, Verhoeven-Duif NM, van Solinge WW, Nieuwenhuis EE, et al. Dried blood spot metabolomics reveals a metabolic fingerprint with diagnostic potential for Diamond Blackfan Anaemia. *British Journal of Haematology* **193** (2021) 1185–1193. doi:10.1111/BJH.17524.

[11] Sharma A, Vans E, Shigemizu D, Boroevich KA, Tsunoda T. DeepInsight: A methodology to transform a non-image data to an image for convolution neural network architecture. *Scientific Reports* **9** (2019) 11399. doi:10.1038/s41598-019-47765-6.

[12] Culley C, Vijayakumar S, Zampieri G, Angione C. A mechanism-aware and multiomic machine-learning pipeline characterizes yeast cell growth. *Proceedings of the National Academy of Sciences of the United States of America* **117** (2020) 18869–18879. doi:10.1073/pnas.2002959117.

[13] Alghamdi N, Chang W, Dang P, Lu X, Wan C, Gampala S, et al. A graph neural network model to estimate cell-wise metabolic flux using single-cell RNA-seq data. *Genome Research* **31** (2021) 1867–1884. doi:10.1101/GR.271205.120.

[14] Wang L, Nie R, Yu Z, Xin R, Zheng C, Zhang Z, et al. An interpretable deep-learning architecture of capsule networks for identifying cell-type gene expression programs from single-cell RNA-sequencing data. *Nature Machine Intelligence* **2** (2020) 693–703. doi:10.1038/s42256-020-00244-4.

[15] Wang L, Miao X, Nie R, Zhang Z, Zhang J, Cai J. MultiCapsNet: A General Framework for Data Integration and Interpretable Classification. *Frontiers in Genetics* **12** (2021) 2237. doi:10.3389/fgene.2021.767602.

[16] Pai S, Hui S, Isserlin R, Shah MA, Kaka H, Bader GD. netDx: interpretable patient classification using integrated patient similarity networks. *Molecular Systems Biology* **15** (2019) e8497. doi: 10.15252/MSB.20188497.

[17] Costello Z, Martin HG. A machine learning approach to predict metabolic pathway dynamics from time-series multiomics data. *npj Systems Biology and Applications* **4** (2018) 1–14. doi: 10.1038/s41540-018-0054-3.

[18] Koh HW, Fermin D, Vogel C, Choi KP, Ewing RM, Choi H. iOmicsPASS: network-based integration of multiomics data for predictive subnetwork discovery. *npj Systems Biology and Applications* **5** (2019). doi:10.1038/S41540-019-0099-Y.

[19] Zhang X, Xing Y, Sun K, Guo Y. OmiEmbed: A Unified Multi-Task Deep Learning Framework for Multi-Omics Data. *Cancers* **13** (2021) 3047. doi:10.3390/CANCERS13123047.

[20] Bahado-Singh RO, Sonek J, McKenna D, Cool D, Aydas B, Turkoglu O, et al. Artificial intelligence and amniotic fluid multiomics: prediction of perinatal outcome in asymptomatic women with short cervix. *Ultrasound in Obstetrics & Gynecology* **54** (2019) 110–118. doi:10.1002/UOG.20168.

[21] Nguyen ND, Jin T, Wang D. Varmole: a biologically drop-connect deep neural network model for prioritizing disease risk variants and genes. *Bioinformatics* **37** (2021) 1772–1775. doi: 10.1093/BIOINFORMATICS/BTAA866.

[22] Leitner M, Fragner L, Danner S, Holeschofsky N, Leitner K, Tischler S, et al. Combined metabolomic analysis of plasma and urine reveals AHBA, tryptophan and serotonin metabolism as potential risk factors in Gestational Diabetes Mellitus (GDM). *Frontiers in Molecular Biosciences* **4** (2017) 84. doi:10.3389/FMOLB.2017.00084.

[23] Hoehenwarter W, Larhlimi A, Hummel J, Egelhofer V, Selbig J, Van Dongen JT, et al. MAPA distinguishes genotype-specific variability of highly similar regulatory protein isoforms in potato tuber. *Journal of Proteome Research* **10** (2011) 2979–2991. doi:10.1021/PR101109A.
